# Particle-Size-Fractionated Coal Gasification Slag as a Supplementary Cementitious Material: Hydration Products, Microstructure Evolution, and Mechanical Performance via Classified Grinding

**DOI:** 10.3390/ma19173736

**Published:** 2026-09-02

**Authors:** Meng Su, Can Chen, Meiqing Chen, Peinian Wang, Nan Ding, Hua Lei, Zhenyun Cheng, Bo Fu

**Affiliations:** College of Civil Engineering, North Minzu University, Yinchuan 720021, China; 20247578@stu.nmu.edu.cn (M.S.); 20247581@stu.nmu.edu.cn (C.C.); 20223644@stu.nmu.edu.cn (M.C.); t20234165@163.com (P.W.); 20247585@stu.nmu.edu.cn (N.D.); 20237539@stu.nmu.edu.cn (H.L.); 2016035@nmu.edu.cn (Z.C.)

**Keywords:** coal gasification slag, particle classification, coal gasification slag powder, ordinary portland cement, mechanical grinding, pozzolanic activity

## Abstract

To promote the high-value utilization of coal gasification slag (CGS) resources and mitigate the environmental issues caused by its accumulation, CGS was separated into five fractions by particle size (2.36–4.75 mm, 1.18–2.36 mm, 0.60–1.18 mm, 0.30–0.60 mm, and 0.15–0.30 mm) and subsequently ground into CGS powders (CGSPs). The physicochemical properties of both CGS and the obtained CGSP were systematically characterized, and the effects of CGSP on the hydration behavior and engineering performance of ordinary Portland cement (OPC) were investigated. The results revealed significant differences in physical properties and composition among the various particle-size fractions of CGS and their corresponding CGSP. When 40 wt.% CGSP was used to replace Portland cement, the C2.36 fraction exhibited the highest early-age compressive strength (16.91 MPa and 25.3 MPa at 3 d and 7 d, respectively), which is attributed to its favorable chemical composition and abundant glassy components. In contrast, the C0.6 fraction achieved the highest 28 d compressive strength (50.0 MPa). The C0.15 fraction showed the lowest strength at all ages, may be mainly due to its high residual carbon content and low reactivity. Overall, the compositional differences among CGS fractions of different particle sizes govern the formation and evolution of hydration products, and the proposed strategy of “classified grinding and quality-oriented utilization” provides an effective approach for the high-value application of CGS in cement-based materials.

## 1. Introduction

China is a typical energy-intensive country characterized by abundant coal resources and limited oil and natural gas reserves, resulting in coal remaining the dominant primary energy source and sustaining extremely high annual consumption levels [1,2]. In recent years, against the backdrop of the strategic adjustment of China’s energy structure, modern coal chemical industries centered on coal gasification technology have developed rapidly [3]. Coal gasification is a clean coal utilization technology in which coal undergoes a series of complex chemical reactions with gasifying agents under high-temperature or high-pressure conditions, resulting in the conversion of coal into gaseous products and a small amount of solid residue [4]. Coal gasification slag (CGS) is the main solid waste generated during this process, which can be classified into coarse slag and fine slag based on its physical form [5]. During coal gasification, the mineral matter in coal is transformed into molten slag. A portion of this molten slag adheres to the inner wall of the gasifier under high-temperature conditions, flows downward along the wall in a molten state, and is rapidly quenched and solidified at the bottom of the reactor, forming coarse slag with relatively large particle sizes, typically light brown or light black in color. In contrast, another portion of the molten slag is entrained by the gas flow and carried out of the reactor together with the syngas into subsequent purification processes, forming fine slag with relatively smaller particle sizes, which is generally dark black due to its high carbon content [6]. In terms of chemical composition, fine slag contains a lower content of basic oxides and a higher content of acidic oxides compared with coarse slag [7]. With the widespread application of coal gasification technology, the generation of gasification slag has increased year by year. According to statistics from the China Coal Processing and Utilization Association, the annual production of gasification slag in China has exceeded 33 million tons [8]. At present, the predominant disposal methods of CGS remain stockpiling and landfilling, and large-scale resource utilization pathways have yet to be established. As a result, the accumulated slag not only occupies substantial land resources but also causes severe environmental pollution and groundwater contamination, posing significant threats to ecological sustainability and attracting widespread concern from both academia and industry [9,10,11,12]. Nowadays, the utilization of CGS in construction materials is considered an effective approach for its resource recovery and for promoting the development of a circular economy.

Cement, particularly ordinary Portland cement (OPC), is an indispensable bulk construction material in modern society, with extremely high global annual production that underpins infrastructure development worldwide [13]. However, the large-scale production of cement is associated with substantial energy consumption and significant carbon emissions. Therefore, considerable efforts have been devoted in the academic community to partially replace cement with industrial solid wastes, aiming to promote sustainable development across economic, social, and environmental dimensions [14,15,16,17].

CGS exhibits potential pozzolanic reactivity, making it a viable partial substitute for cement in cementitious matrixes. Incorporating CGS not only reduces raw material costs and natural resource mining, but also significantly enhances the late-age strength and durability of concrete under appropriate replacement levels [18]. Liu Kaiping et al. reported that incorporating ground coarse and fine gasification slag into concrete produced distinct effects on mechanical properties. Compared with the reference concrete, the concrete containing ground coarse gasification slag exhibited significantly enhanced compressive strength, with a continuous increase in strength as the curing age was prolonged [19]. Zhao Yongbin et al. found that CGS is rich in amorphous glassy phases and can be used as a supplementary cementitious material in concrete, promoting cement hydration and enhancing strength development [20]. Li et al. reported that fine CGS contains a relatively high amount of residual carbon, which may hinder the cementitious reactions between gasification slag and cement or lime. In contrast, coarse slag is enriched in reactive mineral phases, which is beneficial for enhancing the strength of mortar [21]. Wu Hui et al. found that mechanical grinding can induce a “reactive powder effect” of minerals in cementitious materials, thereby enhancing their reactivity [22].

In summary, CGS can be utilized as a substitute for cement and concrete aggregates after appropriate treatment and thus applied in the construction sector [23]. Mechanical activation is a commonly used approach to enhance the reactivity of CGS. However, most previous studies have focused on the use of mixed-grinding CGS, while systematic investigations into the intrinsic differences among different particle-size fractions remain limited. This lack of targeted understanding restricts performance optimization and weakens the development of application-oriented utilization strategies. In this context, the present study aims to classify CGS into distinct particle-size fractions and subsequently grind each fraction into CGS powders (CGSP) with comparable fineness. The microstructural characteristics and their effects on the performance of ordinary Portland cement (OPC) are systematically compared. This work proposes a “classified grinding and quality-oriented utilization” strategy, providing a new theoretical basis for the high-value utilization of CGS.

## 2. Experimental and Methods

### 2.1. Experimental Materials

**Figure 1:**
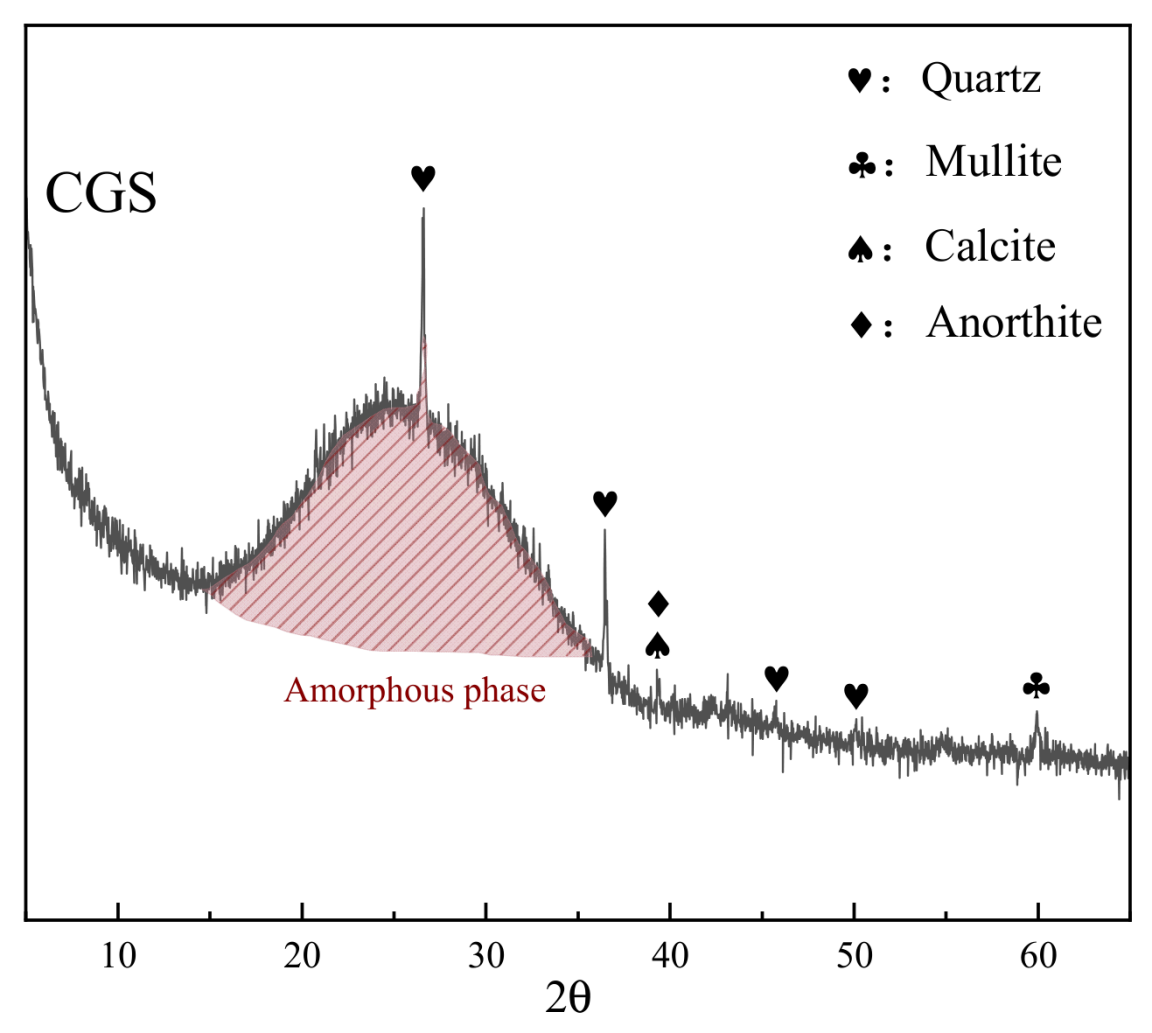
XRD pattern of CGS.

**Figure 2:**
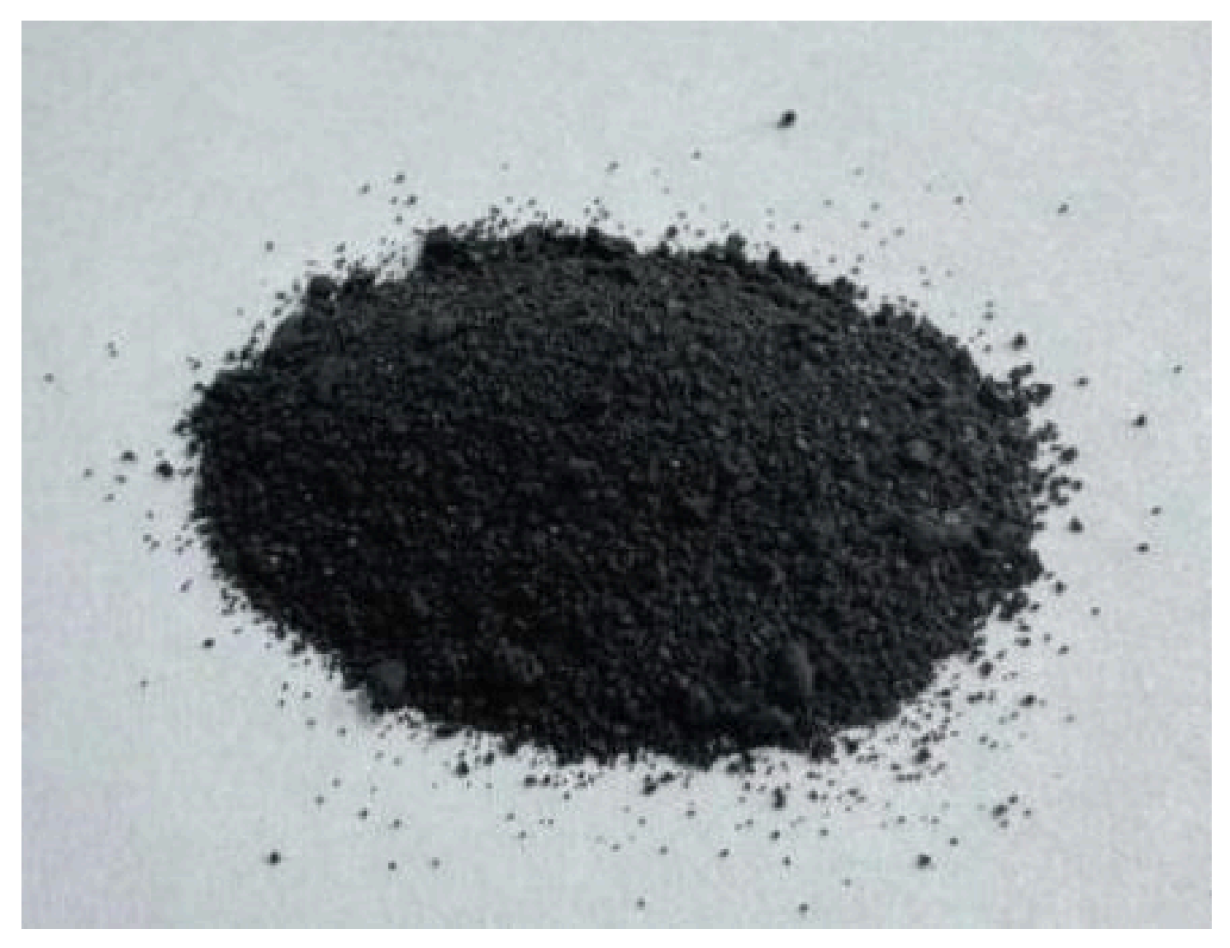
Morphology of CGS.

(1) Cement: Ordinary Portland cement (PO·42.5) was supplied by Ningxia Qingtongxia Cement Co., Ltd. (Qingtongxia, China). The specific surface area was 0.34 m^2^/g, and its main chemical composition is listed in Table 1.
(2) Coal gasification slag: The coal gasification slag was obtained from National Energy Group Ningxia Coal Industry Co., Ltd. (Yinchuan, Ningxia, China). It had a fineness modulus of 1.17. Its main chemical composition is presented in Table 1, while the mineralogical phases and physical morphology are shown in Figure 1 and Figure 2, respectively.
(3) Water: Ultra-pure water was used throughout all experiments.

### 2.2. Experimental Scheme

#### 2.2.1. Raw Material Preparation

A vibrating sieve shaker (STSJ-3A three-dimensional tap sieve shaker, Zhejiang Tuo Gong Instrument Manufacturing Co., Ltd., Shaoxing, Zhejiang, China) was used to separate the CGS. The sieving frequency was set at 1300 rpm with a sieving duration of 10 min. The slag was sieved into five particle-size fractions: 2.36–4.75 mm, 1.18–2.36 mm, 0.60–1.18 mm, 0.30–0.60 mm, and 0.15–0.30 mm. It should be noted that each designation represents a particle-size range rather than a single discrete particle size. For convenience in subsequent analysis, these fractions are abbreviated throughout the manuscript, including tables, figures, figure captions, and discussion, as 2.36 mm, 1.18 mm, 0.6 mm, 0.3 mm, and 0.15 mm, respectively.

**Figure 3:**
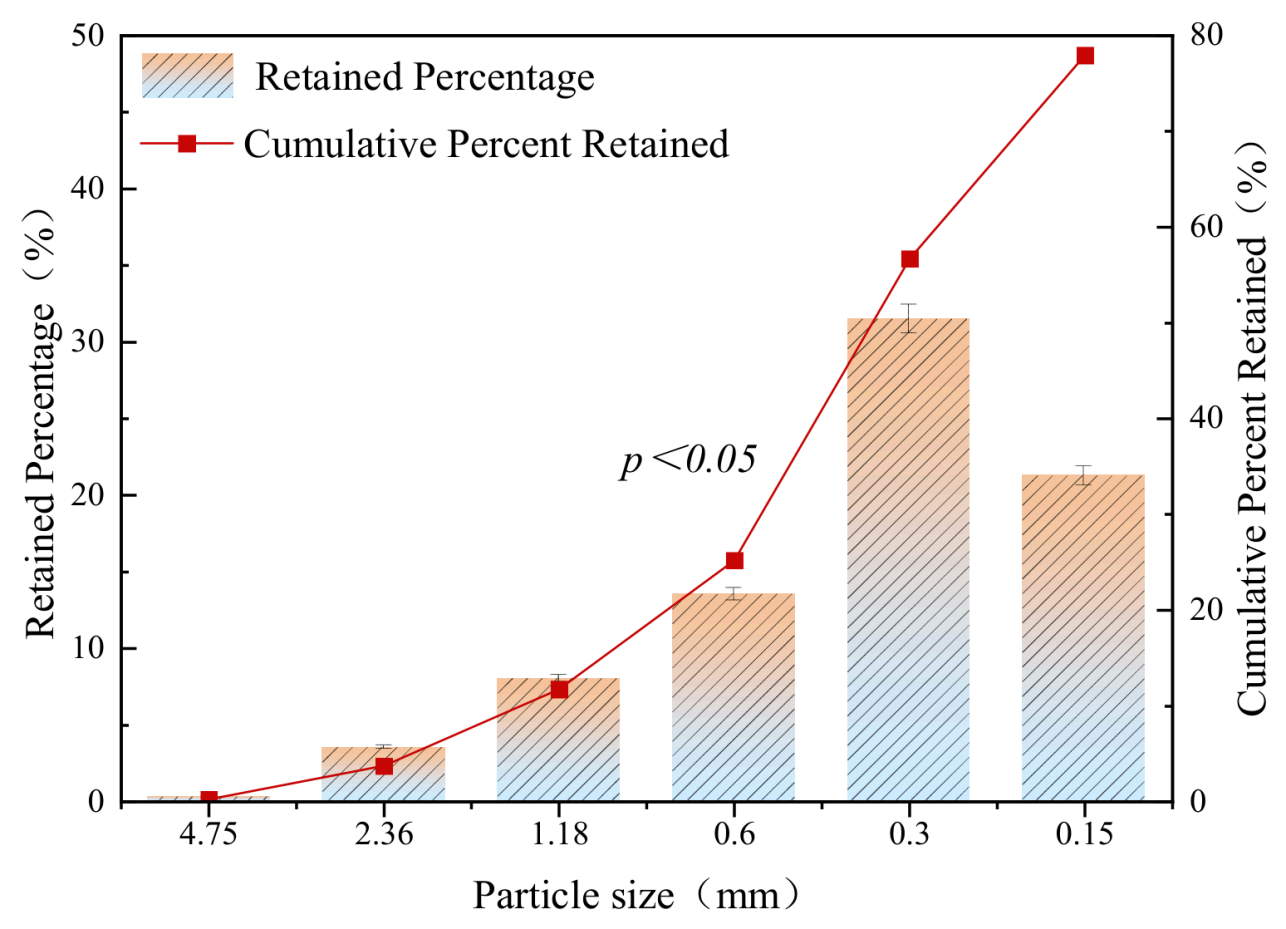
Sieving results of CGS.

The retained mass of each particle-size fraction after sieving is shown in Figure 3. As shown in Figure 3, the retained mass of CGS first increases and then decreases with decreasing particle size, reaching a maximum at 0.30 mm. Equal masses of each fraction were subsequently washed repeatedly with flowing clean water until no visible fine particles were released. The samples were then dried in a vacuum drying oven at 100 °C for 24 h.

**Figure 4:**
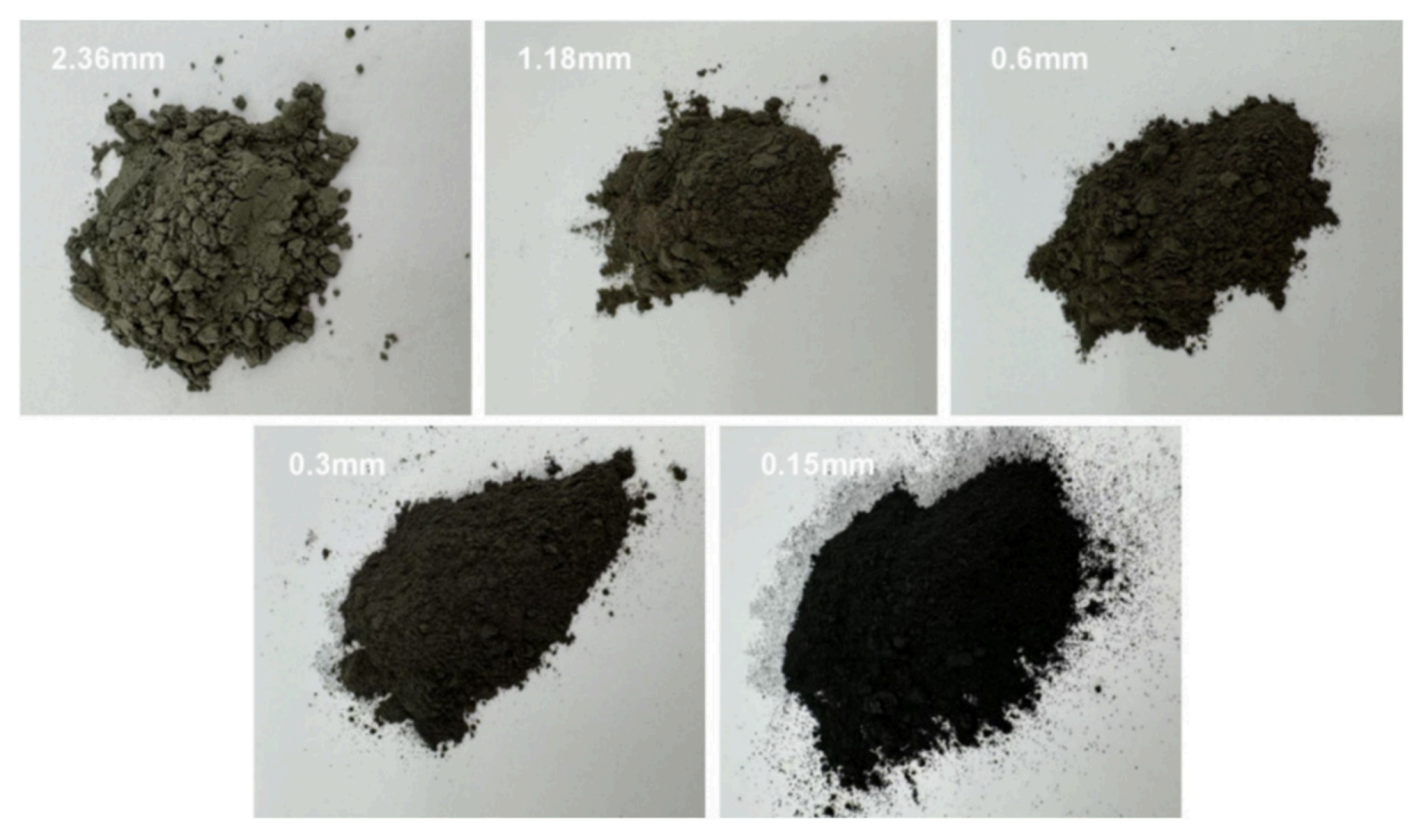
Morphology of fractionated CGSP.

Mechanical activation was performed using a ball mill (GMS5-2, Changsha Miqi Instrument Equipment Co., Ltd., Changsha, China) on each washed and oven-dried CGS fraction until a constant mass was achieved. The milling parameters were set as follows: operating frequency of 50 Hz, power of 0.75 kW, and voltage of 220 V, with a rotational speed controlled at 350–400 rpm. The grinding duration was 5 h. Steel balls with diameters of 2–10 mm were used as grinding media. A mass ratio of 500 g CGS to 2000 g steel balls was adopted, and the materials were loaded into a 5 L steel milling jar for mechanical activation [24]. After grinding, the powders were sieved through a 200-mesh sieve to obtain coal gasification slag powder (CGSP). The physical morphology of the fractionated CGSP samples is shown in Figure 4.

**Figure 5:**
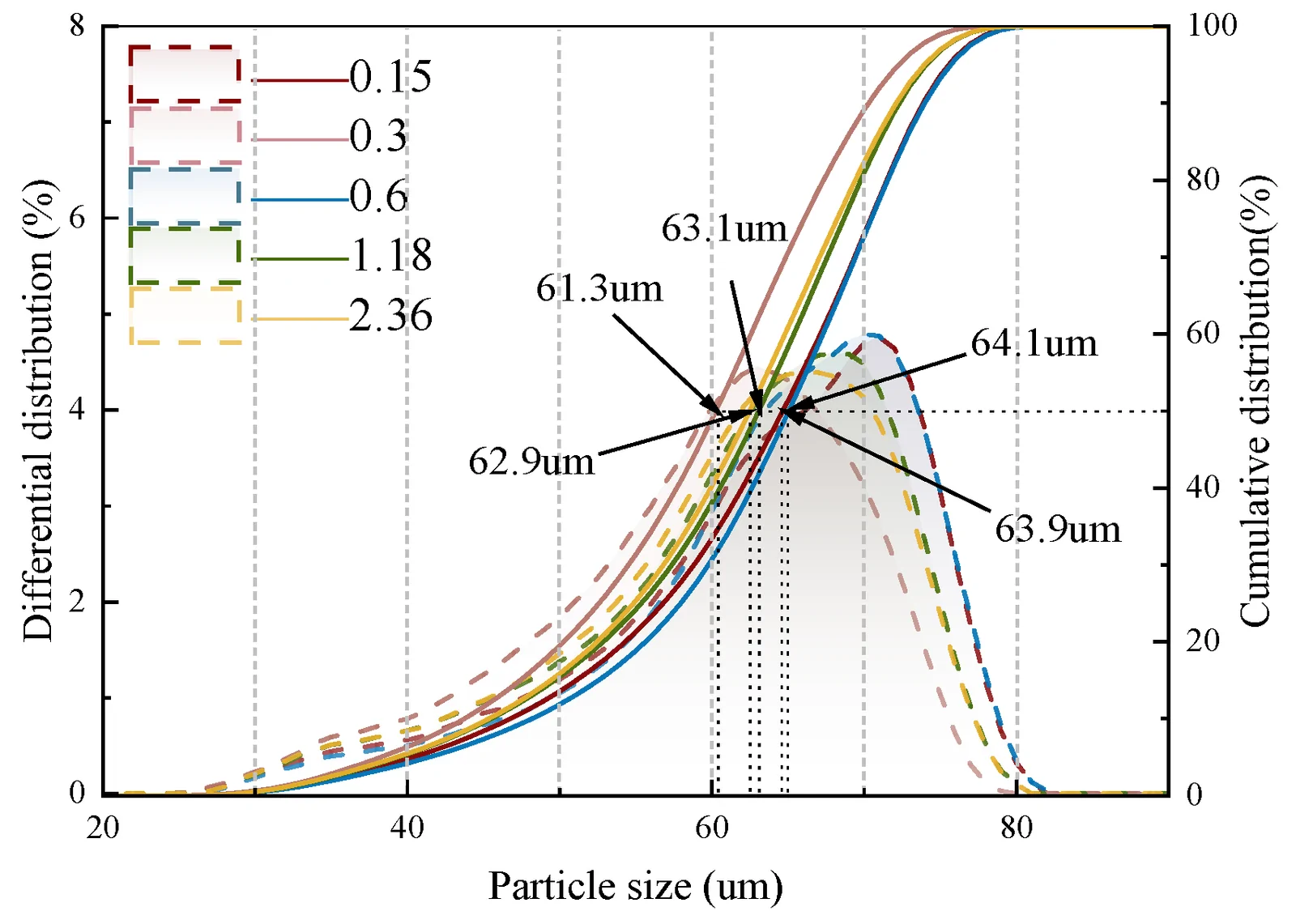
Particle size distribution curves of fractionated CGSP.

The particle size distribution and specific surface area of the obtained CGSP were measured using a laser particle size analyzer (Bettersize2000, Dandong Bettersize Instruments Co., Ltd., Dandong, China). The particle size distribution curves are shown in Figure 5, and the specific surface area results are summarized in Table 2. As shown in Figure 5 and Table 2, the particle size of all CGSP samples was mainly distributed in the 60–70 μm range, and the specific surface area ranged from 0.25 to 0.35 m^2^/g. These results indicate that mechanical grinding substantially reduced the differences in fineness among CGSP fractions, resulting in powders with broadly comparable fineness. Nevertheless, some variation in specific surface area remained. Therefore, the residual differences in fineness may have contributed to variations in hydration and strength development, although the inherent physicochemical heterogeneity of the original CGS fractions remained an important factor influencing their performance.

#### 2.2.2. Specimen Preparation and Curing

In this study, cubic specimens with dimensions of 40 mm × 40 mm × 40 mm were prepared for compressive strength testing. The water-to-binder ratio was fixed at 0.36. The control group was prepared using pure OPC and designated as C0. The other mixtures incorporated 40 wt.% CGSP with different particle-size fractions replacing OPC, and were labeled as C2.36, C1.18, C0.6, C0.3, and C0.15, respectively. The detailed mix proportions are presented in Table 3. After casting, all specimens were placed in a standard curing chamber for curing periods of 3 d, 7 d, and 28 d. Upon reaching the designated curing ages, the compressive strength was measured using a compression testing machine. A CGSP replacement level of 40 wt.% was selected to introduce a relatively high proportion of industrial solid waste while enabling the performance differences associated with different CGSP particle-size fractions to be evaluated under the same mixture conditions. Since only one replacement level was investigated, the conclusions regarding the particle-size effects are limited to the 40 wt.% CGSP replacement condition and should not be generalized to other replacement levels.

**Figure 6:**
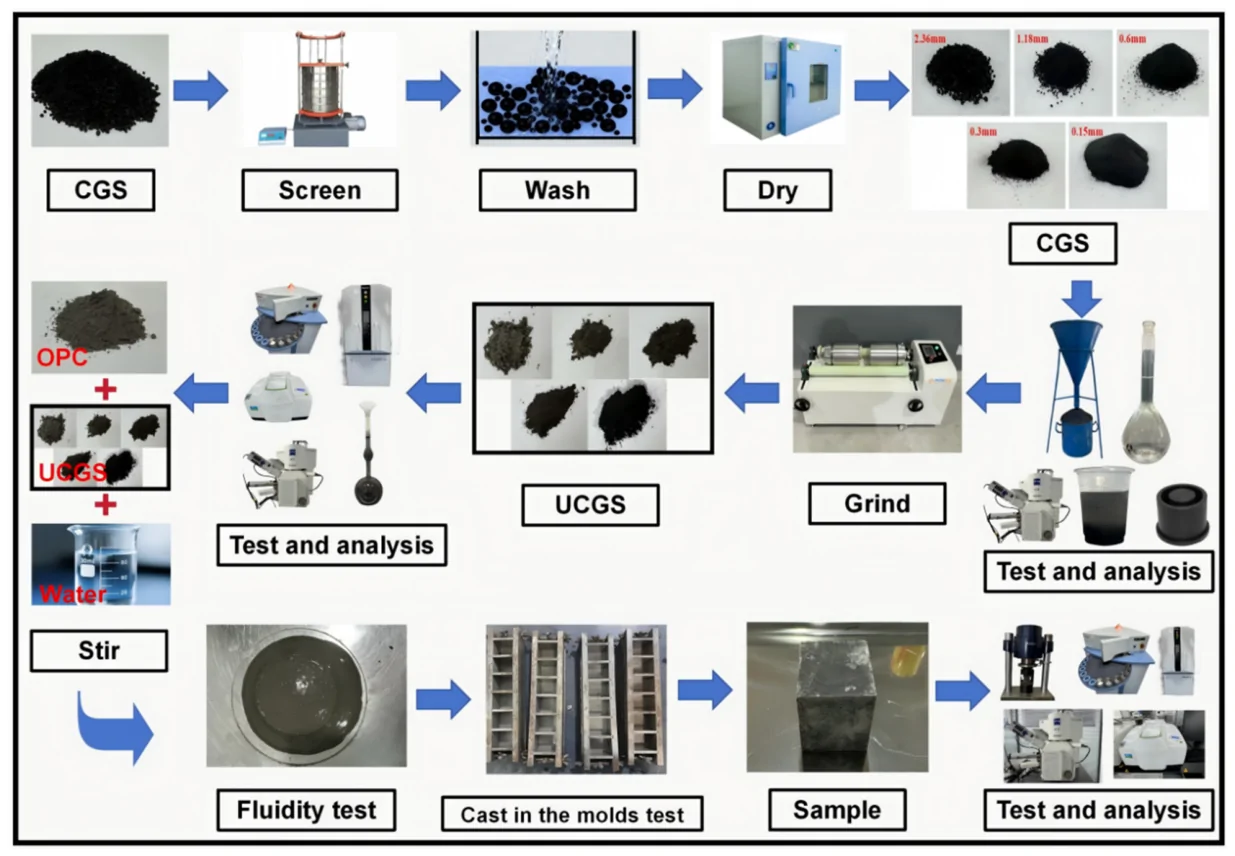
Experimental procedure.

The specimens cured to the designated ages were immersed in absolute ethanol for more than 72 h to terminate further hydration. Subsequently, the samples were dried in a vacuum oven at 40 °C for at least 10 h. The dried specimens were then either ground into powder or retained in bulk form for subsequent microstructural characterization. The detailed experimental procedure is shown in Figure 6.

#### 2.2.3. Testing Methods

Crushing Value Test of CGS: The crushing value of each CGS particle-size fraction was determined according to Method T 0350–2005 of the Test Methods of Aggregate for Highway Engineering (JTG 3432–2024 [25]). For each fraction, a 330 g sample was placed into the fine aggregate crushing value mold and loaded at a rate of 500 N/s to 25 kN. After maintaining the load for 5 s, the load was released. The crushed material was then sieved using the lower-limit sieve of the corresponding particle-size fraction, and the masses of the retained and passing materials were measured to calculate the crushing value. Three parallel specimens were tested for each fraction, and the arithmetic mean was taken as the final result.

Fluidity test: The fluidity of the cement paste was measured using a truncated cone mold. The mold had an upper diameter of 30 mm, a lower diameter of 60 mm, and a height of 60 mm. Before testing, the surface of the flow table and the inner wall of the mold were wiped with a wet cloth to ensure a moist condition. The freshly mixed paste was then poured into the mold. After lifting the mold, the spread diameter of the paste was recorded within 30 s. The average value of two perpendicular measurements was taken as the fluidity of each mixture [26].

Setting time test: The initial and final setting times of the fresh cement paste were determined using a Vicat apparatus in accordance with GB/T 1346-2011 [27]. The reported results were calculated as the average of two repeated measurements.

Compressive strength test: Cubic paste specimens measuring 40 × 40 × 40 mm were prepared, and their compressive strength was measured after 3 d, 7 d, and 28 d of curing. The loading procedure followed the principle of compressive strength testing specified in GB/T 17671–2021 [28], while the specimen geometry was modified according to the cement paste system investigated in this study. The same specimen dimensions and testing procedure were used for all mixtures to ensure comparability. For each mixture and curing age, six specimens were tested. The arithmetic mean of the six measurements was initially calculated. If one value deviated from the mean by more than ±10%, it was excluded and the mean of the remaining five values was recalculated. If another value still exceeded ±10% of the recalculated mean, the set of results was considered invalid. After excluding outliers, the mean of at least four valid measurements was used as the final compressive strength. All results are reported as mean ± standard deviation (SD).

XRD analysis: X-ray diffraction (XRD) was performed using a SmartLab SE X-ray diffractometer (Rigaku Corporation, Tokyo, Japan). The step size was set to 0.02°, the scanning rate was 1°/min, and the scanning range was 5–65°.

FTIR analysis: Fourier transform infrared (FTIR) spectra were recorded using a Nicolet iS50 spectrometer (Thermo Fisher Scientific, Waltham, MA, USA). The scanning range was 4000–400 cm^−1^, with a spectral resolution of ≤0.0 9 cm^−1^ and 32 accumulative scans. Prior to testing, both the samples and potassium bromide (KBr) were thoroughly dried. The sample powder was then mixed with KBr at a mass ratio of 1:100 and ground uniformly in an agate mortar, followed by pellet preparation. Functional groups and chemical bonds were identified based on the absorption of infrared radiation by molecular vibrations [29].

TG-DTG analysis: Thermogravimetric-differential thermogravimetric (TG-DTG) measurements were conducted using an STA 449 F3 simultaneous thermal analyzer (Netzsch Instrument GmbH, Selb, Germany) to evaluate the thermal mass loss of the cement group and the CGSP-blended group at 3 d and 28 d. The temperature ranged from 30 to 1000 °C, with a heating rate of 10 °C/min. Nitrogen was used as the purge gas, and argon was used as the protective gas [4].

SEM-EDS analysis: Scanning electron microscopy coupled with energy-dispersive X-ray spectroscopy (SEM-EDS) was performed using a Sigma 500 field-emission scanning electron microscope (Carl Zeiss, Oberkochen, Germany) to observe the microstructures of CGS particles with different particle sizes, CGSPs, and specimens cured for 3 and 28 d. Prior to observation, the sample surfaces were sputter-coated with gold to ensure adequate electrical conductivity [4].

Data analysis: All macroscopic performance tests were conducted with at least three independent replicates to ensure the reproducibility of the results. The results are presented as mean ± standard deviation (SD). Differences among mixtures were evaluated using one-way analysis of variance (ANOVA) followed by Tukey’s post hoc test, with *p* < 0.05 considered statistically significant.

## 3. Results and Discussion

### 3.1. Physicochemical Properties of CGS

#### 3.1.1. Characteristics of Classified CGS Particles

Table 4 presents the measured bulk density, apparent density, packing porosity, water absorption, and LOI of CGS with different particle sizes. It can be observed that significant differences exist in these physical parameters among the various particle-size fractions.

In terms of bulk density, the results showed that the bulk density of CGS first decreased and then increased with decreasing particle size, reaching a maximum value of 1.35 g/cm^3^ at 0.30 mm. For particles in the 0.6–2.36 mm range, the relatively large and irregular morphology tends to form a stable “arching structure” through interparticle interlocking, leading to higher void content and lower bulk density. The high bulk density at 0.30 mm may be attributed to the moderate particle size, which provides favorable flowability and packing ability; additionally, the particle self-weight is sufficient to overcome interparticle friction, enabling dense packing under gravity [30]. With a further decrease in particle size to 0.15 mm, the particle self-weight decreases significantly while surface adhesion forces become more pronounced, promoting agglomeration and reducing flowability, thus hindering further densification and resulting in a decline in bulk density.

The apparent density of CGS showed only minor variation among different particle sizes, indicating that the true density is not significantly affected by particle size. However, the packing porosity exhibited a clear dependence on particle size. The 0.30 mm fraction had the lowest packing porosity (46.22%), further confirming its densest particle packing. In contrast, the porosity of the 0.15 mm fraction increased sharply to 52.96%, which may be due to the agglomeration of fine particles caused by increased specific surface energy; the resulting loose agglomerates introduce a large number of internal voids within the packing structure.

The water absorption of CGS continuously increased with decreasing particle size. This trend can be attributed to two main factors. On the one hand, the reduction in particle size leads to a substantial increase in specific surface area, providing more active sites for water adsorption and enabling the binding of water molecules through physical adsorption and hydrogen bonding. On the other hand, as indicated by the LOI results, the loss on ignition increases with decreasing particle size, which may suggest a higher residual carbon content in finer fractions. The residual carbon, owing to its porous structure and hydrophilic nature, further enhances the water absorption capacity of the finer fractions [3,31].

**Figure 7:**
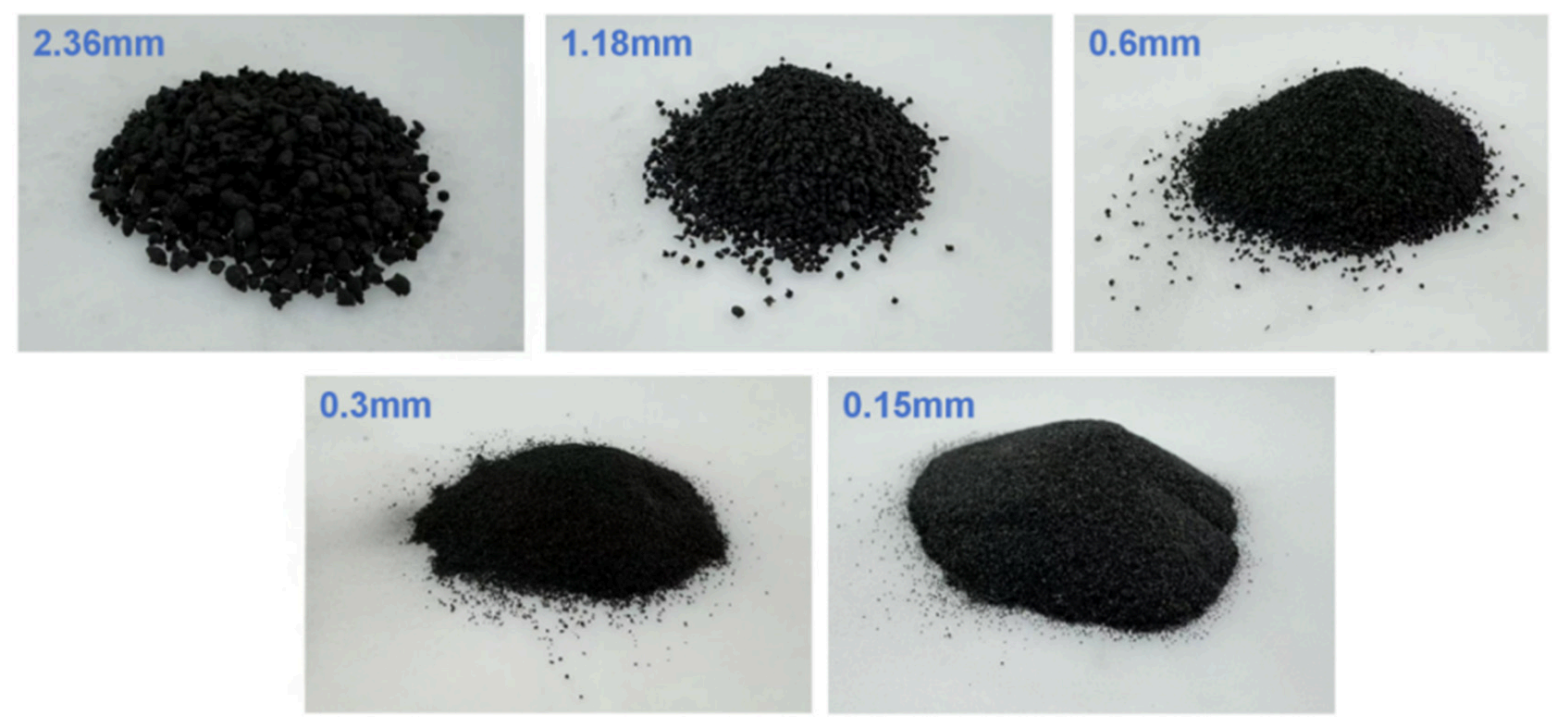
Morphology of CGS with different particle sizes.

**Figure 8:**
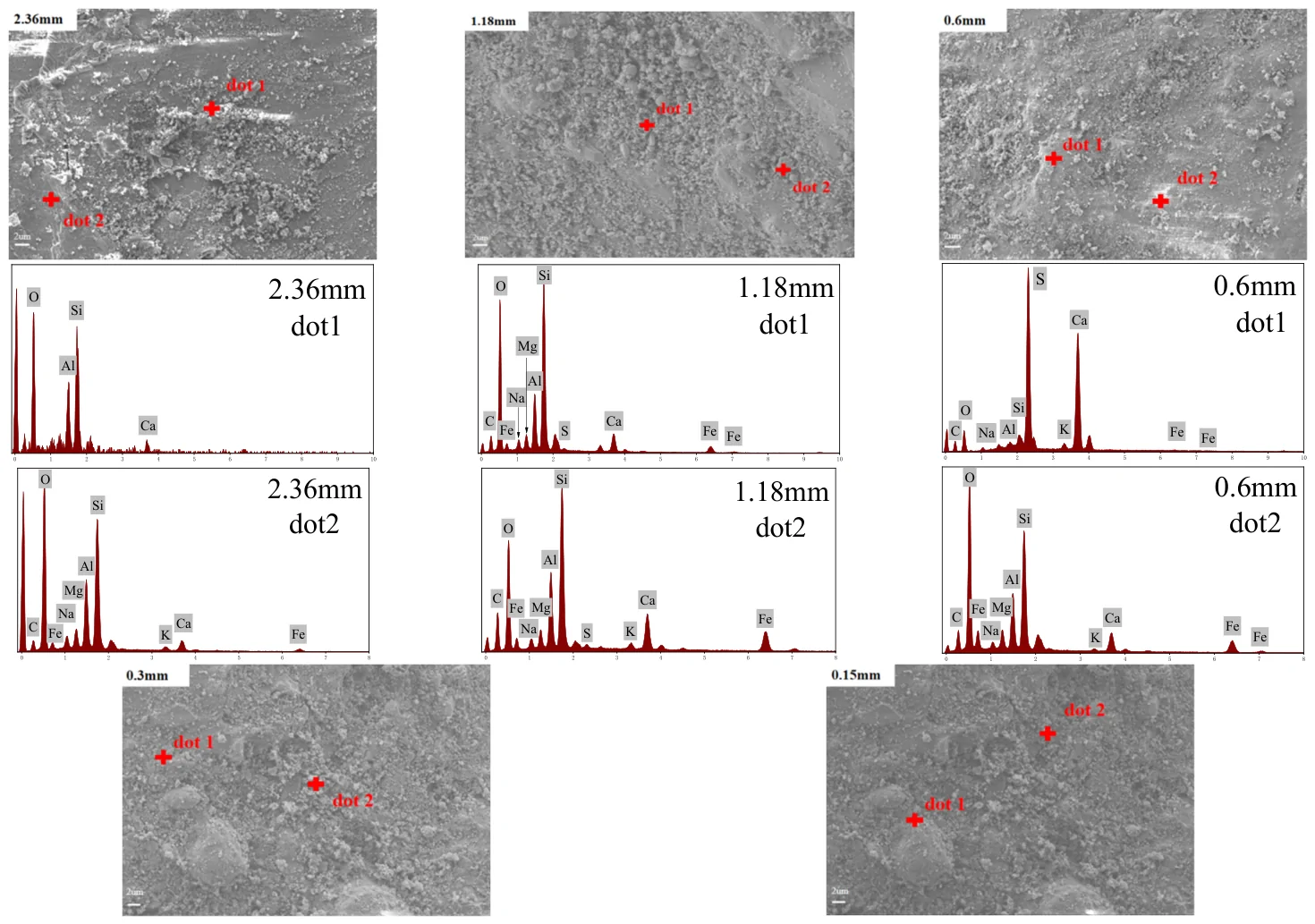

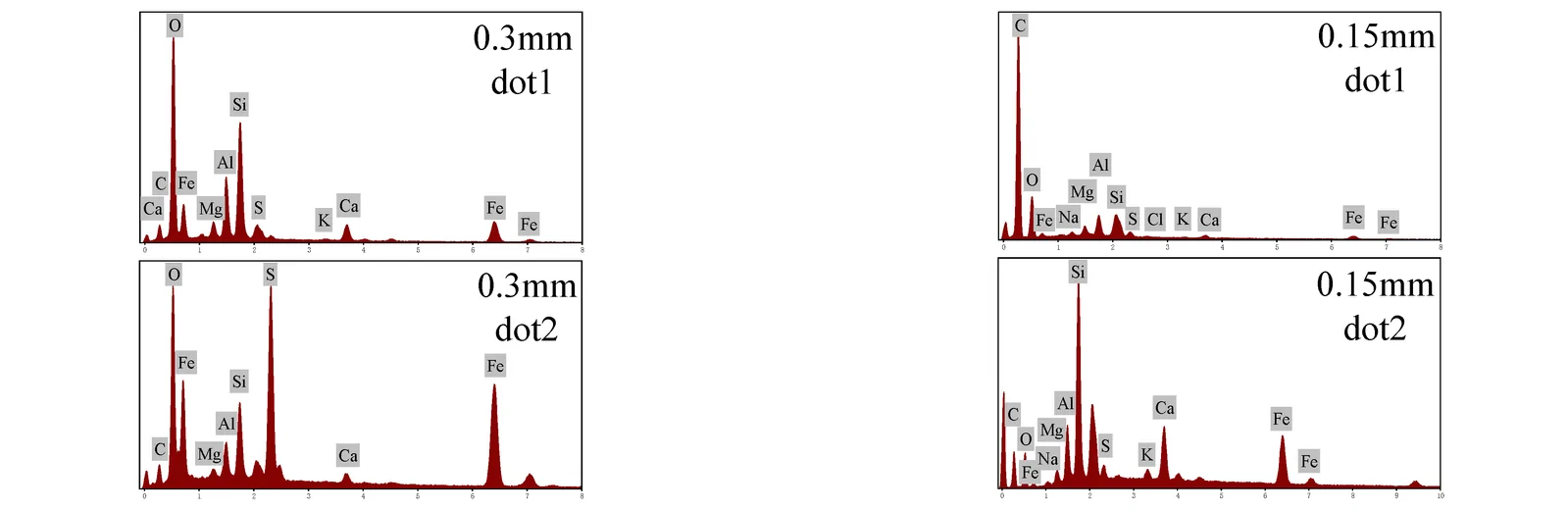
SEM images and EDS spectra of CGS with different particle sizes.

Figure 7 shows the physical morphology of CGS with different particle sizes, while Figure 8 presents the corresponding SEM-EDS comparison results. It can be observed that the microstructural characteristics of CGS vary with particle size. The coarse fractions (0.6–2.36 mm) exhibit pronounced morphological heterogeneity, with particles predominantly existing in flaky and blocky forms. The particle surfaces are highly heterogeneous, with well-developed fissures. Locally, obvious glaze-like textures and glass microspheres formed by the condensation of molten phases can be observed [7]. In contrast, the fine fraction (0.15–0.30 mm) tends to exhibit more spherical or ellipsoidal particle shapes. The particle surfaces are covered by a large number of isolated or aggregated fine spherical particles and fragmented flocculent materials, presenting a typical matte, rough texture [7,30]. Such relatively regular geometries facilitate particle sliding and packing, thereby improving the bulk density.

EDS analysis revealed significant compositional heterogeneity among different CGS particle-size fractions [32]. The 2.36 mm and 1.18 mm fractions mainly exhibited Si-Al-rich glassy phases with variable Ca distribution, whereas localized calcite-rich regions were observed in the 1.18 mm fraction. The 0.3 mm fraction showed evident Fe enrichment, indicating the accumulation of iron-bearing minerals, while the finest fraction (0.15 mm) exhibited a pronounced C signal associated with high residual carbon content [33]. These results demonstrate that particle-size separation effectively differentiates the distribution of reactive glassy phases and inert mineral components in CGS.

**Figure 9:**
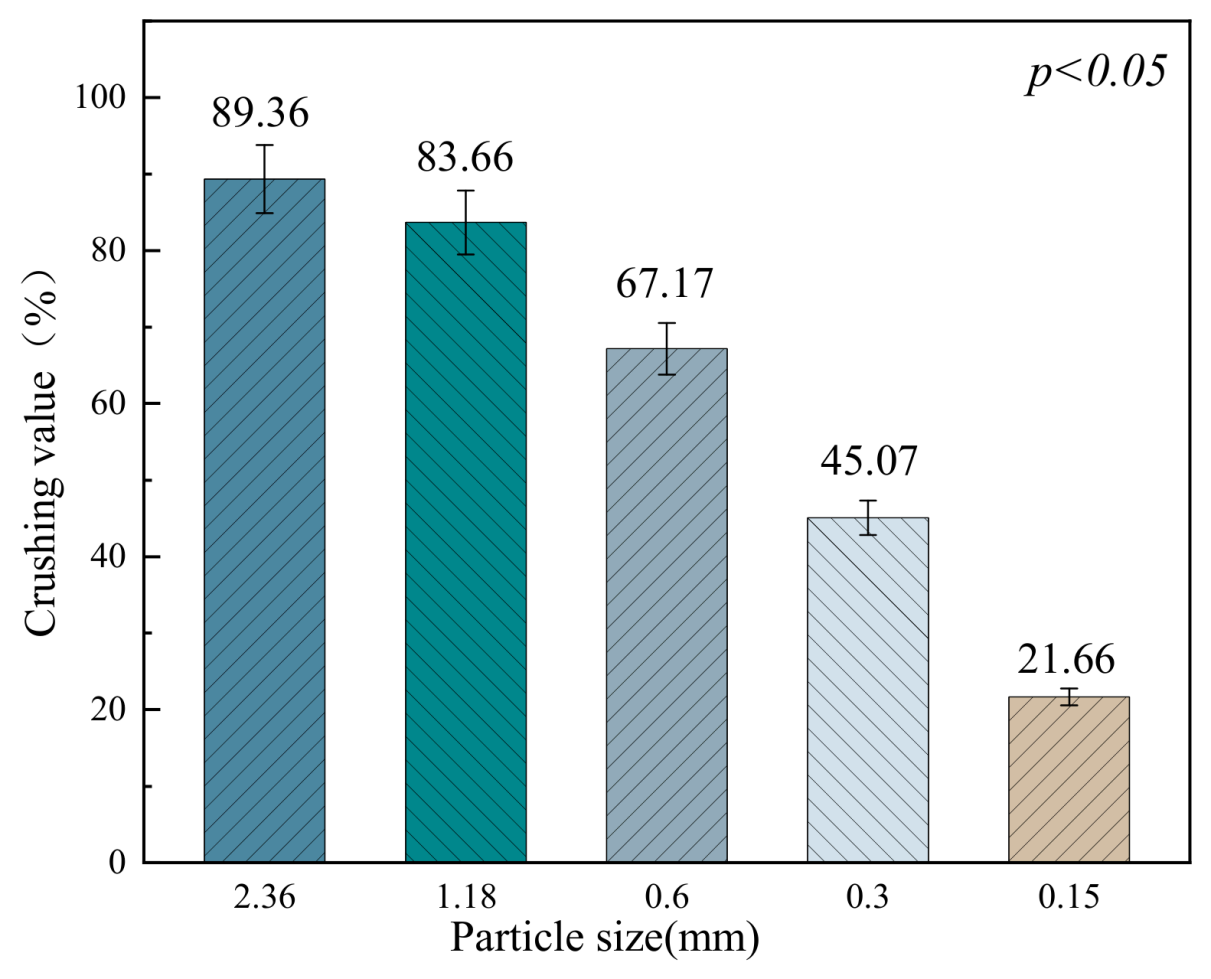
Crushing value of CGS with different particle sizes.

Figure 9 shows the crushing index of CGS with different particle sizes. The results indicate that the crushing value decreases with decreasing particle size, which is consistent with the findings of Xu et al. [33]. It can be observed that the 2.36 mm fraction exhibits the highest crushing value, approximately 89.36%. Combined with the SEM observations (Figure 8), this fraction shows a typical honeycomb-like or foam-like porous structure. The black elongated textures distributed on the particle surfaces are actually microcracks induced by thermal stress during the high-temperature cooling process of the slag [7]. These inherent microdefects are more widely distributed in larger particles, leading to stress concentration under loading and consequently structural failure, which is reflected in a high crushing value [34]. From a processing perspective, a high crushing value indicates easy fragmentation, that is, better grindability, which can be beneficial for ball milling. It can be observed from Figure 8 that with decreasing particle size, the microstructure of CGS gradually evolves from a porous and irregular morphology toward a more compact and regular structure [35]. The 0.15–0.30 mm fraction exhibits particles with clearer edges, smoother surfaces, and significantly reduced internal porosity. Such structural continuity and densification effectively reduce defect sensitivity under mechanical loading.

#### 3.1.2. Characteristics of Fractionated CGSP

Table 5 presents the chemical composition results of CGSPs with different particle sizes. It can be seen that the inorganic components of CGS are mainly composed of SiO_2_, Al_2_O_3_, CaO, and Fe_2_O_3_, with minor amounts of MgO, K_2_O, and Na_2_O also present [36]. We can observe compositional differences among the different fractions. Coarse particles (2.36 mm) exhibited relatively higher Al_2_O_3_ contents, whereas intermediate fractions (1.18–0.6 mm) were enriched in SiO_2_. The finest fraction (0.3–0.15 mm) showed the highest Fe_2_O_3_ content, which may suggest the preferential enrichment of iron-bearing phases during particle classification.

**Figure 10:**
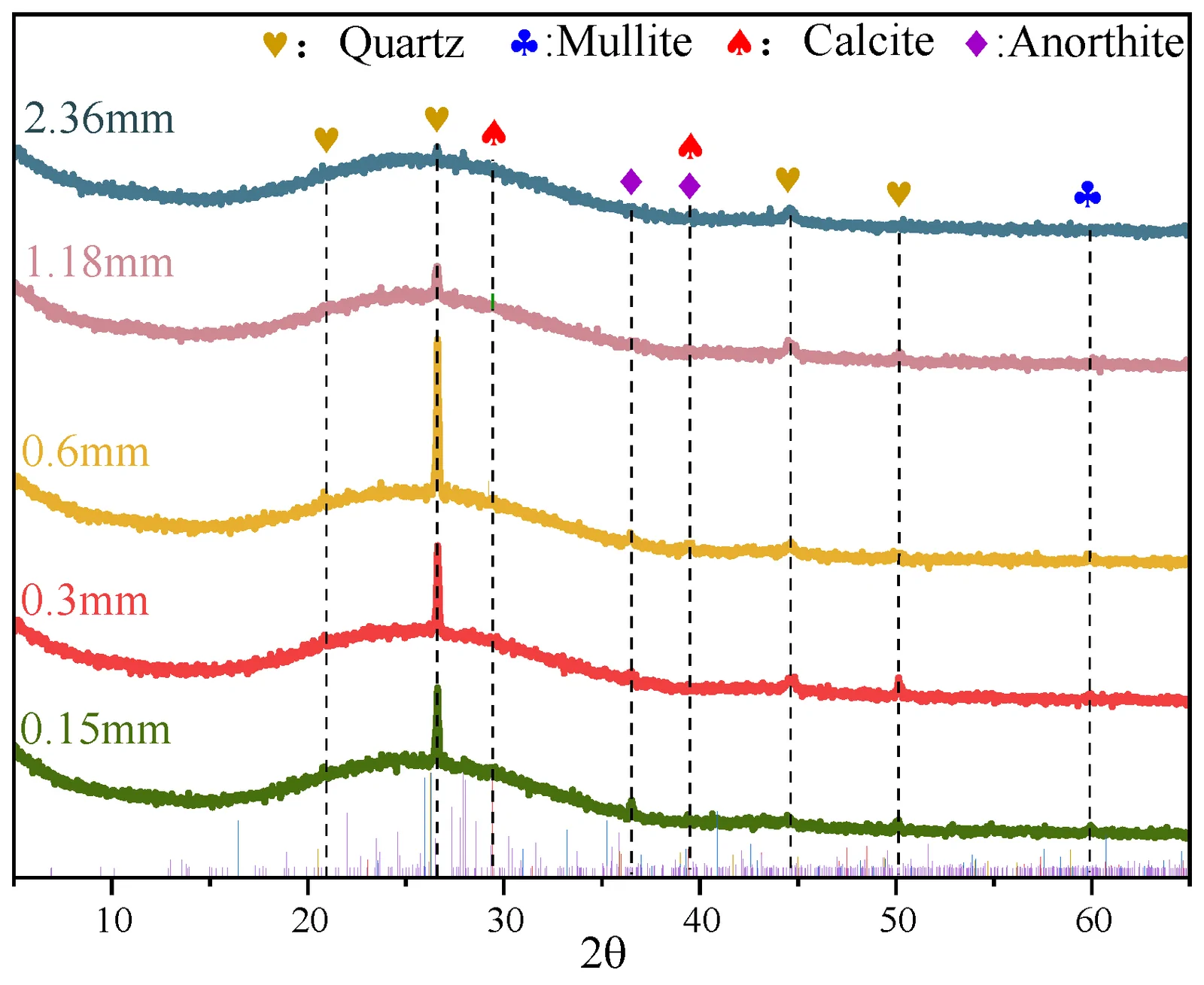
XRD patterns of fractionated CGSP.

The XRD patterns of fractionated CGSP are shown in Figure 10. All samples exhibited an obvious “broad hump” centered at approximately 26° (2θ) within the range of 20–30° (2θ), which may indicate the presence of a large amount of amorphous phases in the CGS, including amorphous residual carbon and aluminosilicate glass phases [37]. In terms of crystalline minerals, the phase evolution of the different fractions can be summarized as follows: the crystalline phases of particles larger than 1.18 mm were mainly composed of quartz, calcite, and anorthite, whereas the characteristic peaks of mullite appeared and gradually intensified in particles smaller than 1.18 mm [38].

The quartz diffraction peaks initially intensified and then weakened with decreasing CGS particle size, indicating the relative enrichment of quartz in intermediate fractions, while its lower abundance in coarse fractions may result from dilution by other mineral phases or weathering effects. In contrast, the continuously enhanced mullite peaks suggest its preferential accumulation in finer fractions. Although calcite is thermally unstable at high temperatures, it remained detectable in CGS, probably due to the short residence time during gasification or subsequent carbonation/recrystallization of carbonate phases. The calcite content exhibited a similar non-monotonic variation, increasing first and then decreasing with particle size reduction [7,36,39]. Anorthite is formed through the reaction between mullite and calcium aluminate phases generated from the thermal decomposition of calcite in coal at approximately 1200 °C, and it tends to disappear at around 1400 °C [40]. The variation in anorthite content with particle size was similar to that of quartz.

**Figure 11:**
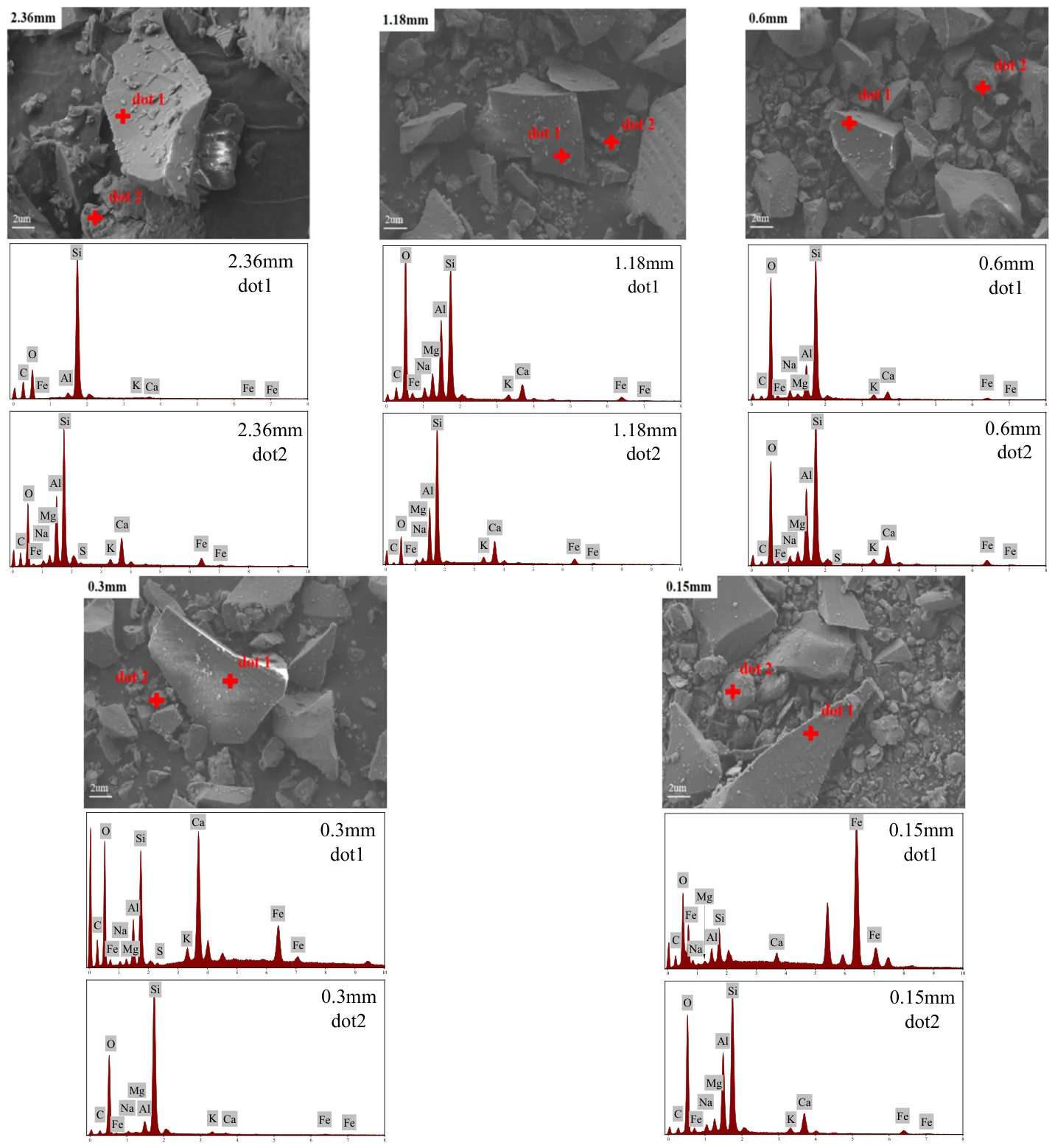
SEM–EDS of fractionated CGSP.

Figure 11 shows SEM-EDS images of the CGSP after ball milling. The particles were angular and irregular in shape. EDS analysis showed that mechanical milling did not significantly alter the elemental composition of the slag across different particle sizes, compared with the untreated sample. However, within the milled powder, the iron content was lower in larger particles than in smaller ones [41]. This trend was particularly evident in the 0.3 mm and 0.15 mm size fractions. The results suggest that iron increased the grinding resistance of the coal gasification slag [38].

**Figure 12:**
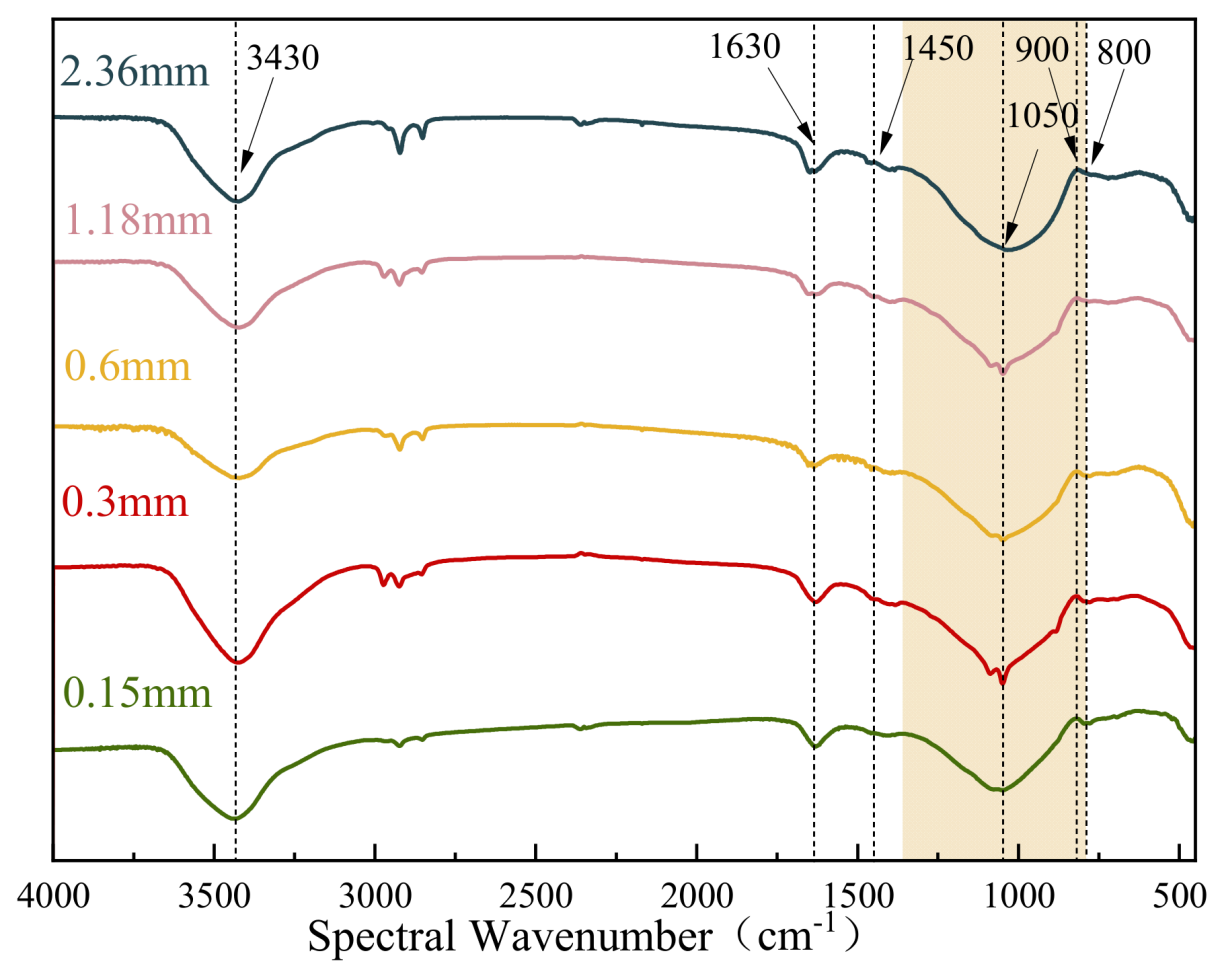
FTIR spectra of fractionated CGSP.

Figure 12 shows the FTIR spectra of fractionated CGSP. The absorption peaks at 3430 cm^−1^ and 1630 cm^−1^ are associated with the stretching and bending vibrations of O-H, respectively. The peak at 1450 cm^−1^ corresponds to the asymmetric stretching vibration of C-O. The peak at 1050 cm^−1^ originates from the asymmetric stretching vibration of Si-O-Si (Al), while the peak at 800 cm^−1^ is attributed to the symmetric stretching vibration of Si-O. The shoulder peak at 900 cm^−1^ is assigned to the stretching vibration of Si-O [42,43,44].

The FTIR spectra indicate evident variations in mineral phases and glass-network structures among different particle-size fractions. The 2.36 mm fraction exhibited a weak Si-O symmetric stretching band at 800 cm^−1^ but a pronounced shoulder peak at 900 cm^−1^, suggesting a relatively high abundance of disrupted Si-O bonds and non-bridging oxygen (NBO) in the amorphous aluminosilicate phase [42]. This structure may provide more reactive sites during hydration. In contrast, the strong carbonate-related band at 1450 cm^−1^ in the 1.18 mm fraction is consistent with the presence of carbonate phases, consistent with the higher calcite content observed by XRD. The 0.6 mm fraction showed the strongest Si-O vibration at 800 cm^−1^, corresponding to a higher quartz content. The enhanced 3430 cm^−1^ band in the 0.3 mm fraction suggests increased surface hydroxyl groups or adsorbed water, while the weakened 1050 cm^−1^ band in the 0.15 mm fraction indicates a lower contribution of reactive glass phases, possibly due to dilution by iron oxides and residual carbon [42,43,44].

**Figure 13:**
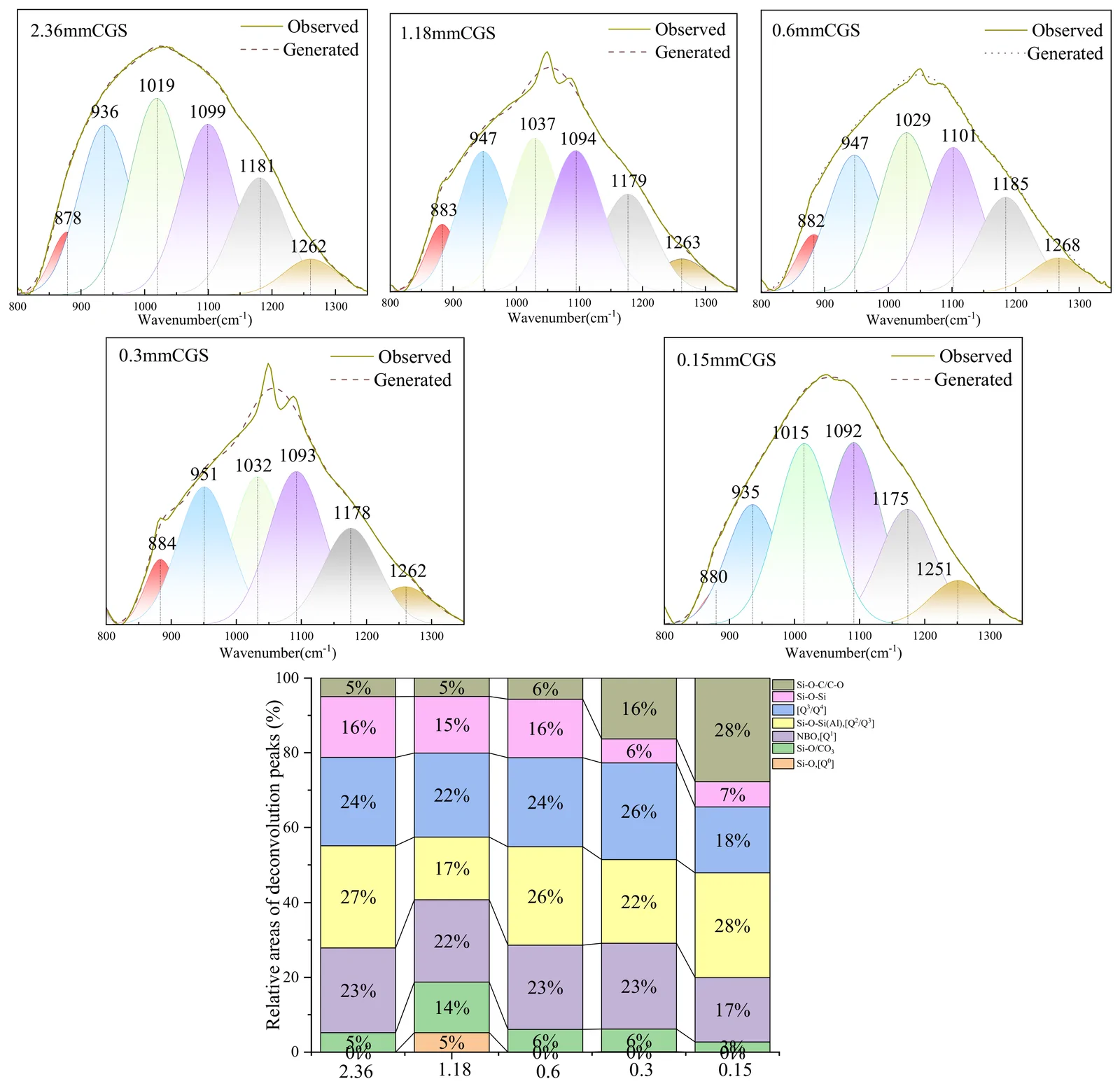
Relative area of deconvoluted peaks in the 800–1350 cm^−1^ range.

To further evaluate the polymerization degree of the aluminosilicate network, peak deconvolution was performed in the 800–1350 cm^−1^ region (Figure 13). The fitted spectra provide semi-quantitative information on the distribution of different silicate structural units (Q^n^) and carbon-related functional groups. The band at 820–850 cm^−1^ is mainly associated with symmetric Si-O stretching vibrations, corresponding to low-polymerized silicate structures such as Q^0^ units [45]. The absorption band at 870–890 cm^−1^ is primarily attributed to the bending vibration of CO_3_^2−^ groups, with possible contributions from Si-O vibrations of depolymerized silicate structures [46]. The band at 920–950 cm^−1^ is assigned to Si-O stretching vibrations related to NBO, representing broken bonds within the glass network and potential active sites for dissolution [42,47]. The broad band at 1000–1050 cm^−1^ originates from asymmetric Si-O-Si(Al) stretching vibrations and is mainly associated with moderately polymerized Q^2^/Q^3^ units. The band at 1080–1120 cm^−1^ corresponds to highly polymerized silicate structures (Q^3^/Q^4^ units), although contributions from crystalline silicate phases such as quartz cannot be completely excluded [48]. The weak band at 1150–1200 cm^−1^ is related to the asymmetric stretching vibration of Si-O-Si bonds in highly condensed silicate structures [49,50]. In addition, the band at 1250–1280 cm^−1^ is attributed to Si-O-C and C-O stretching vibrations, reflecting oxygen-containing functional groups associated with residual carbon in CGSP [51].

The deconvolution results revealed distinct differences in the Q^n^ distribution and carbon-related structures among different CGSP fractions. The 2.36 mm fraction exhibited the highest NBO content (23%) and abundant [Q^2^/Q^3^] units, which may suggest more active sites within the glass network and enhanced early dissolution capability [47]. Although it also contained a relatively high proportion of [Q^4^] structures, the coexistence of highly polymerized networks and structural defects contributed to its high early-age reactivity. In contrast, the 1.18 mm fraction showed fewer NBO and a higher carbonate-related contribution, which may suggest limited reactive glass phases [51]. The 0.6 mm fraction presented the most balanced Q^n^ distribution, providing an optimal balance between structural stability and dissolution capability, which favored sustained release of reactive Si and Al species during hydration [50]. The 0.3 mm and 0.15 mm fractions exhibited increased carbon-related bands (16% and 28%, respectively), while the 0.15 mm fraction showed insufficient NBO sites despite a high proportion of polymerized units ([Q^2^/Q^3^] + [Q^4^] = 46%), which may indicate that residual carbon and low glass-phase reactivity restricted its cementitious activity [51].

**Figure 14:**
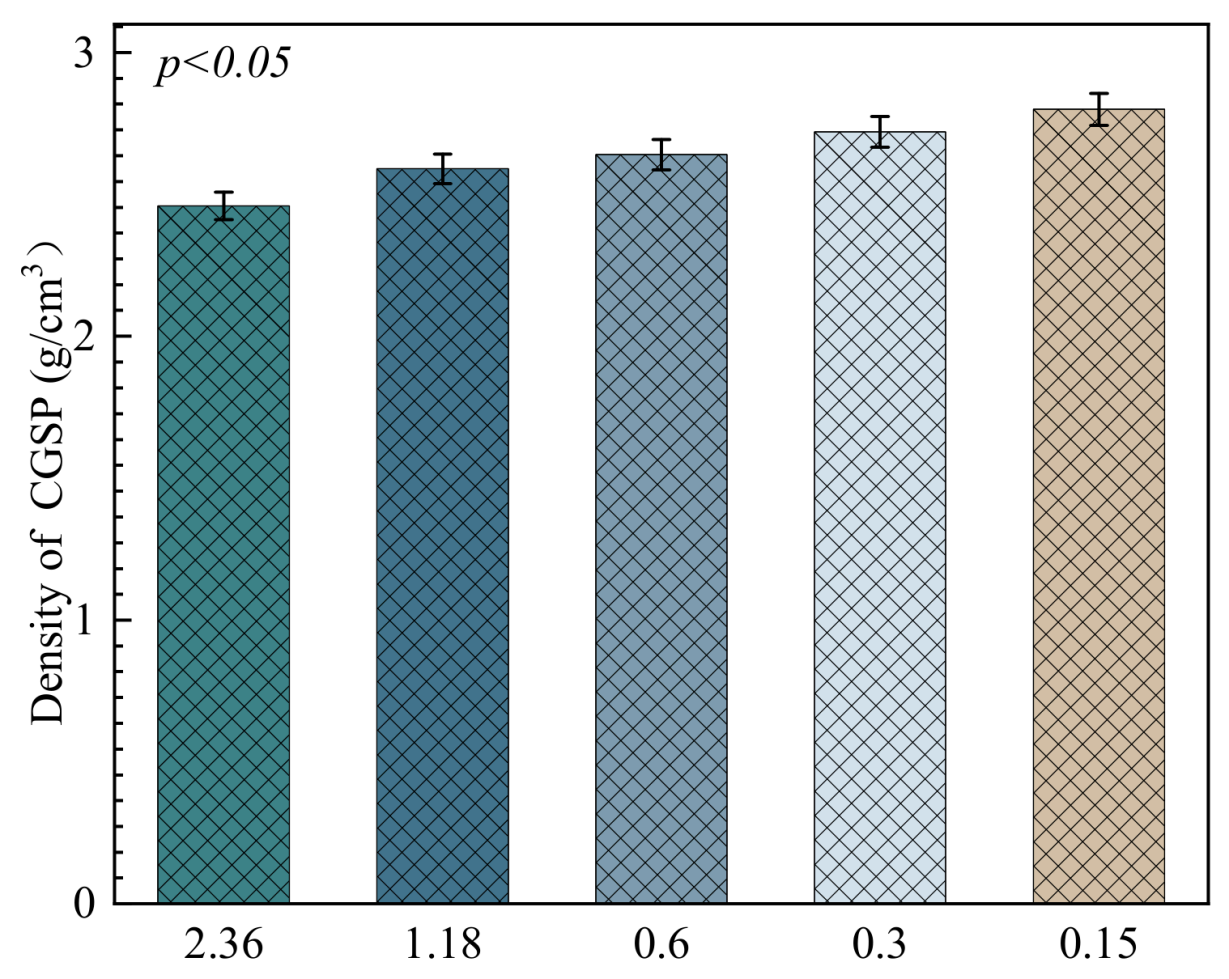
Density of fractionated CGSP.

Figure 14 shows the density variation in CGSPs derived from different CGS fractions. The density increased progressively with decreasing original CGS particle size. The 2.36 mm fraction exhibited the lowest density (2.46 g/cm^3^), which may be attributed to the enrichment of porous aluminosilicate glass phases and relatively low content of dense mineral components. With decreasing particle size, Fe-bearing heavy minerals became enriched, resulting in the highest density (2.80 g/cm^3^) for the 0.15 mm fraction, consistent with the increased Fe_2_O_3_ content observed by XRF [37]. Although residual carbon was also enriched in fine fractions and could decrease density, its effect was outweighed by the accumulation of high-density inorganic phases.

### 3.2. Effects of Fractionated CGSP on OPC Performance

#### 3.2.1. Macroscopic Properties

(1)  Flowability

**Figure 15:**
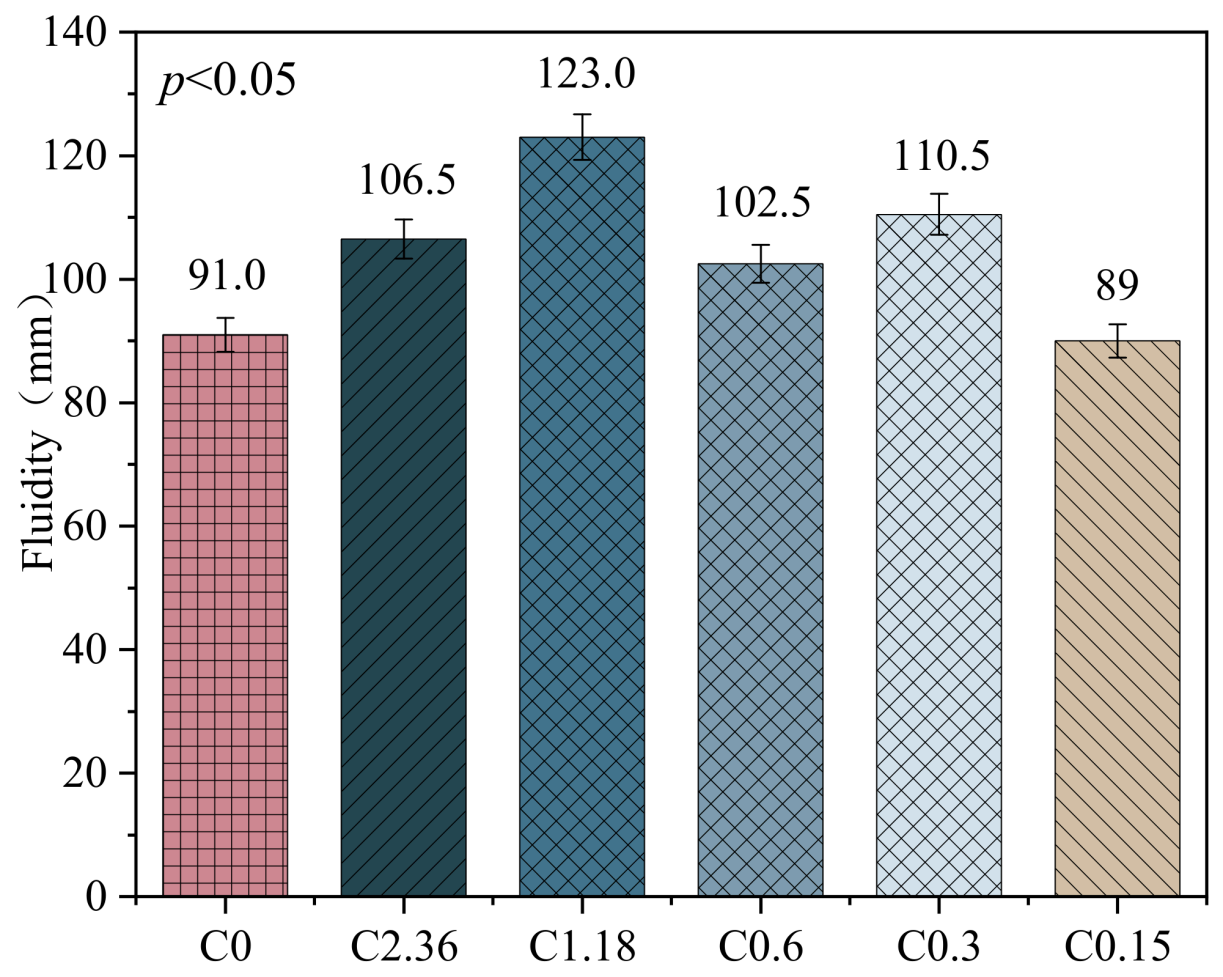
Effect of different CGSP fractions on paste flowability.

Figure 15 illustrates the effect of different CGSP fractions on the fluidity of cement pastes. Compared with the control group (C0) without CGSP, the fluidity of mixtures C2.36, C1.18, C0.6 and C0.3 increased by 17.0%, 35.2%, 12.6% and 21.4%, respectively. In contrast, C0.15 slightly decreased the flowability by 2.2%. The improvement in paste fluidity after CGSP addition can be attributed to two main factors. First, the ultrafine grinding process endows CGSP with a good micro-filling effect. This effect effectively fills the voids between cement particles and releases part of the otherwise entrapped free water [52]. Second, CGSP exhibits relatively lower reactivity than ordinary Portland cement (OPC). Thus, its early-age water demand is reduced, leading to a greater amount of free water in the system [52,53]. However, the C0.15 mixture showed lower fluidity than the C0 group. This is likely due to the relatively high residual carbon content in this CGSP fraction, which increases the water demand and consequently reduces fluidity [37].

As shown in Figure 15, the differences in fluidity among the CGSP fractions may be associated with variations in their chemical compositions and silicate structural characteristics (Table 5 and Figure 13). C1.18 exhibited the highest fluidity, which may be related to its relatively favorable oxide composition and lower water demand. C2.36 also showed improved fluidity, possibly due to its relatively high Al_2_O_3_/SiO_2_ ratio and the particle-filling effect of its mineral components. Although C0.6 had the highest SiO_2_ content, it did not exhibit the highest fluidity, indicating that the total SiO_2_ content alone was not the dominant factor controlling workability. The higher fluidity of C0.3 than C0.6 further suggests that the combined effects of chemical composition, particle packing, and surface characteristics should be considered. In contrast, C0.15 exhibited the lowest fluidity, mainly due to its high loss on ignition, residual carbon content, and water absorption, which reduced the amount of free water available for particle lubrication [37]. FTIR results further indicate differences in the degree of silicate polymerization among the fractions, which may additionally regulate their interactions with water. Overall, the fluidity of CGSP-containing pastes appears to be governed by the combined effects of chemical composition, silicate structural characteristics, particle packing, and water absorption, rather than by any single oxide component.

(2)  Setting Time

**Figure 16:**
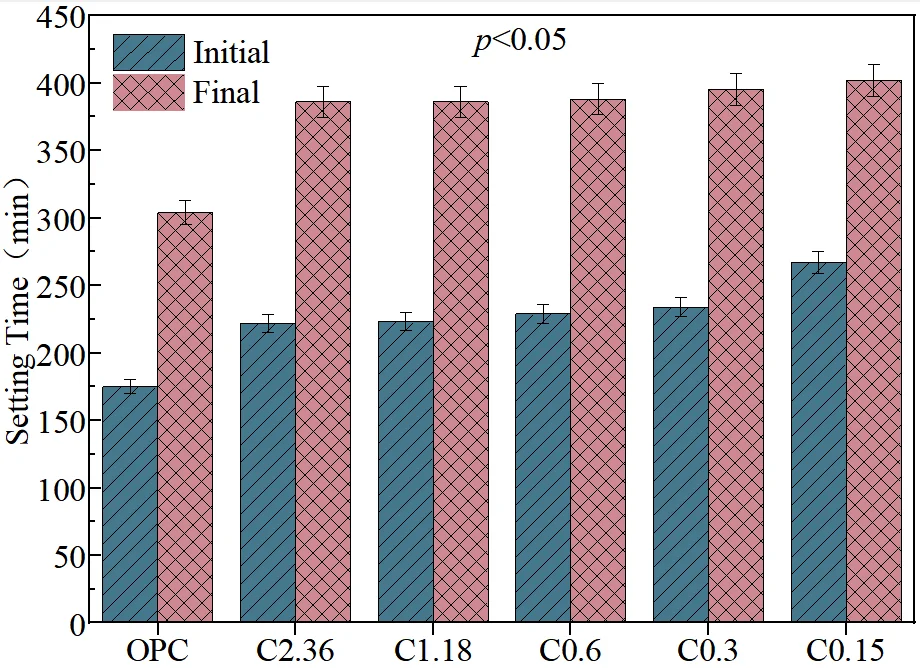
Effect of different CGSP fractions on the setting time of paste.

Figure 16 shows the effect of different CGSP fractions on the setting time of the paste. As can be seen, both the initial and final setting times were significantly prolonged after the incorporation of CGSP. Moreover, the setting time increased monotonically with decreasing particle size of the original CGS. The initial and final setting times of the control group (C0) were 175 min and 304 min, respectively. After the incorporation of CGSP, the initial setting time was extended by 26.9–52.6%, and the final setting time was prolonged by 27.0–32.2%. This retarding phenomenon is mainly attributed to the “dilution effect”, whereby the replacement of part of the cement with CGSP reduces the cement content, thereby directly decreasing the formation rate of early hydration products [54]. Although all CGSP mixtures exhibited longer setting times than the C0 group, some differences were still observed among them, which may be related to variations in their chemical and mineral compositions. Among them, the C2.36 exhibited the fastest setting behavior. This is commonly ascribed to its highest Al_2_O_3_ content, which accelerates the rapid precipitation of early AFt and enables the paste to form a solid structure in a short time [55]. In addition, its lowest Fe_2_O_3_ content may reduce the inert interference during the hydration process. The setting time of the C1.18 was longer than that of the C2.36 mixture. This is probably because the CGSP in this group has a low Al_2_O_3_ content, and part of CaO exists in the form of inert calcite, which restricts the early hydration efficiency of aluminosilicate phases [56]. The C0.6 contains highly polymerized silica-rich glass phases; its slow early dissolution rate may lead to the reduced generation amount of AFt [56]. The C0.3 contains a relatively high proportion of inert mineral phases, which may greatly dilute the cementitious components and affect the setting process of the paste. The C0.15 exhibited the slowest setting behavior, which is likely associated with its highest Fe_2_O_3_ content, the lowest content of active glassy phases, and the physical barrier effect caused by high residual carbon content. These combined factors jointly inhibited the interconnection of hydration products.

(3)  Compressive Strength

**Figure 17:**
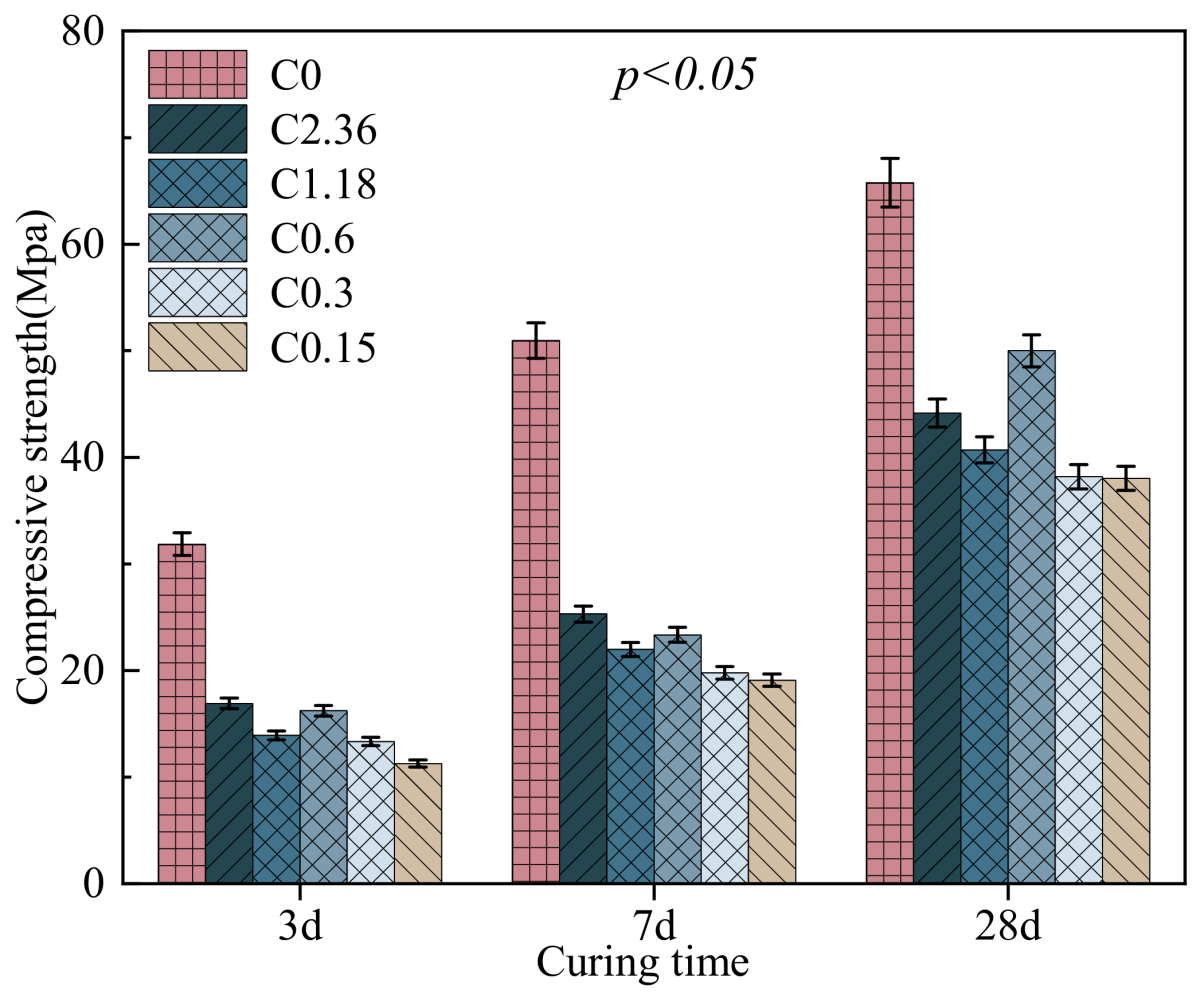
Effect of different CGSP fractions on the compressive strength of specimens.

Figure 17 shows the effect of CGSP with different particle size fractions on the compressive strength of specimens. Overall, the compressive strength decreased significantly after CGSP was incorporated into the system. However, the strength increment of all groups at 28 d was higher than that of the OPC group, indicating that the pozzolanic activity of CGSP with various particle sizes improved the secondary hydration reaction of the matrix [57].

At the early stage, the C2.36 exhibited the best mechanical performance [58], with 3 d and 7 d compressive strengths of 16.91 MPa and 25.3 MPa, respectively, reaching 53.1% and 49.7% of the corresponding strengths of the reference cement group. This may be attributed to its higher content of reactive glass phases and relatively accessible aluminosilicate structures, which may promote the rapid dissolution of Si and Al species and accelerated the formation of C-(A)-S-H gel and AFt [56]. Therefore, C2.36 showed the fastest hydration response and superior early-age strength.

However, the strength development of C2.36 slowed at later ages, resulting in a lower 28 d strength than C0.6. This may be related to the rapid consumption of reactive components during the early hydration stage, which limited further pozzolanic reactions. In contrast, the C0.6 achieved the highest 28 d strength (50.0 MPa), owing to its moderately polymerized silica-rich glass phase. The sustained release of reactive Si species was considered to promote continuous CH consumption and C-S-H formation, leading to a denser hydration structure and improved long-term strength [56].

The C1.18 exhibited moderate strength development. Although it contained abundant Ca-bearing phases, part of Ca existed as relatively inert calcite, limiting its contribution to early hydration. The C0.3 and C0.15 showed poor strength development, which may be due to the lower content of reactive glass phases and enrichment of inert components. In particular, the high residual carbon and Fe-bearing phases in C0.15 may have hindered hydration product formation, potentially contributing to the lowest strength among all mixtures [21,58].

#### 3.2.2. Microscopic Properties

(1)  XRD

**Figure 18:**
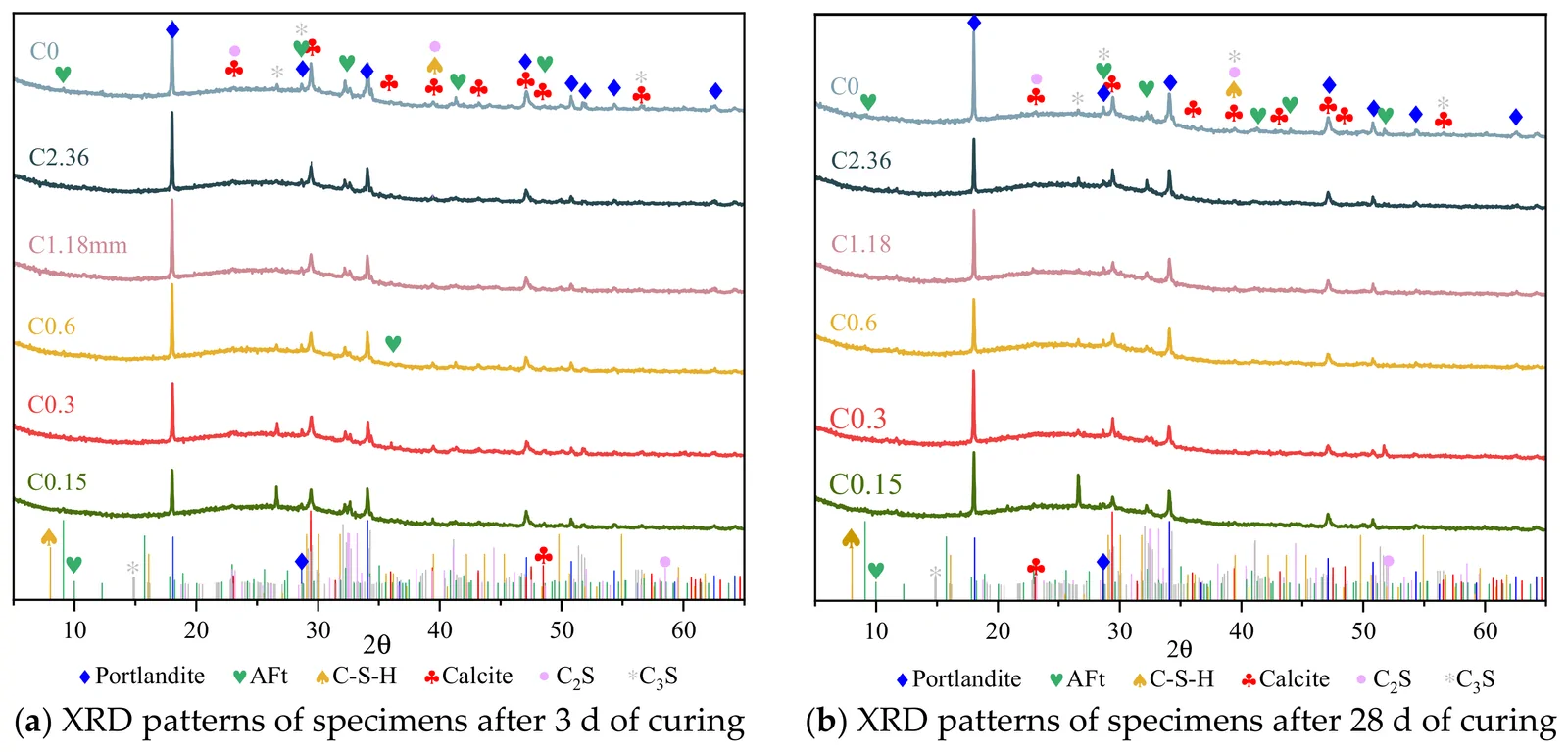
XRD patterns of specimens.

Figure 18 shows the XRD patterns of the reference cement group and the specimens with different CGSP contents after 3 d and 28 d of curing. Phase analysis indicates that the main hydration products in all samples include portlandite (CH), ettringite (AFt), calcium silicate hydrate (C-S-H) gel, calcite (CaCO_3_), as well as partially unhydrated C_2_S and C_3_S, while C-S-H gel is mainly present as an amorphous phase [52].

At 3 d (Figure 18a), all CGSP-blended specimens exhibited weaker CH and AFt diffraction peaks than the reference group, which may be mainly attributed to the dilution effect caused by cement replacement and the relatively slow reaction of CGSP at early ages [24]. Among different fractions, the C2.36 mixture showed stronger AFt and C-S-H-related signals, which may suggest a higher degree of early hydration, which agrees with its superior early-age strength [56]. The C0.6 mixture showed relatively weak AFt formation but detectable C-S-H generation, suggesting that its reactive silica-rich glass phase may have begun to participate in pozzolanic reactions. In contrast, the weak hydration-product peaks and strong residual clinker peaks observed in C0.3 and C0.15 may indicate insufficient activation of reactive phases and limited early hydration.

After 28 d (Figure 18b), the hydration products remained similar, but obvious differences in reaction degree were observed among the mixtures. The C0.6 specimen exhibited the lowest CH diffraction intensity, which may suggest the most extensive consumption of CH through pozzolanic reactions. This promoted continuous C-S-H formation and explains its highest 28 d compressive strength [58]. The C2.36 specimen also showed reduced CH intensity and enhanced hydration-product peaks, confirming its sustained reactivity; however, its lower long-term strength development compared with C0.6 suggests that part of its reactive components was consumed at earlier stages.

The C1.18 mixture showed a noticeable reduction in calcite diffraction intensity after 28 d. This may be associated with the participation of carbonate phases in hydration reactions, where calcite can provide nucleation sites for hydration products and react with aluminate phases to form calcium carboaluminate phases, contributing to pore refinement. However, its relatively lower reactive glass content limited its overall pozzolanic contribution. In contrast, the C0.3 and C0.15 mixtures showed limited CH consumption and weak hydration-product formation, confirming their poor pozzolanic reactivity and corresponding low mechanical performance [24].

(2)  FTIR

**Figure 19:**
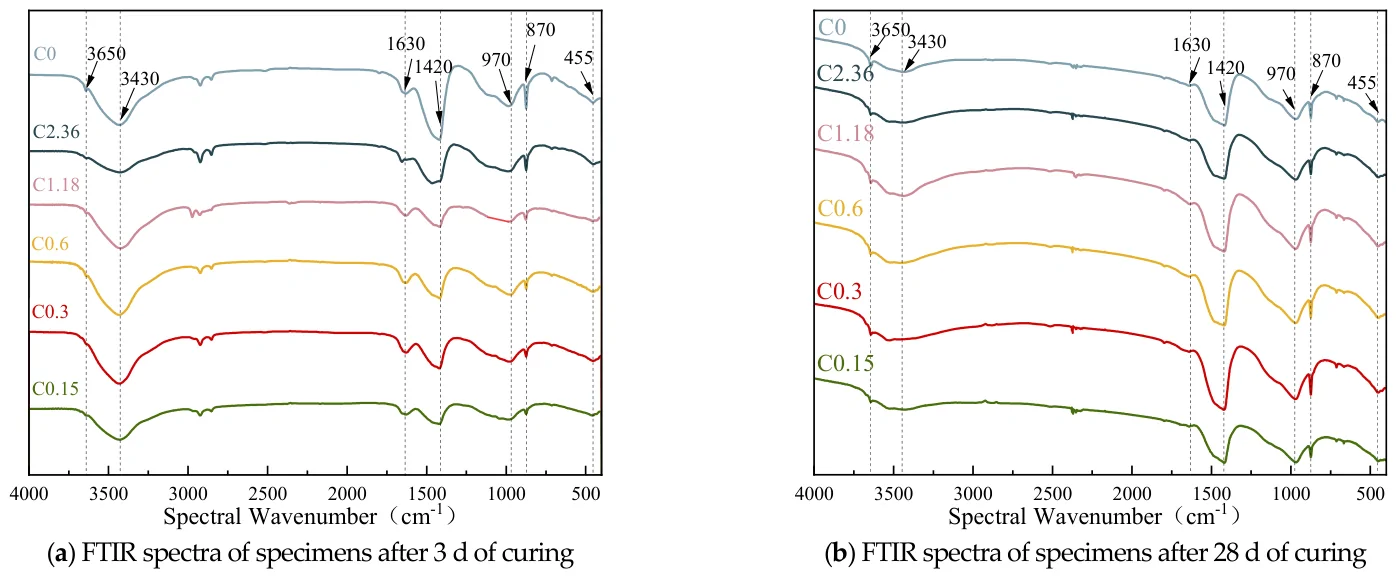
FTIR spectra of specimens.

FTIR spectra were used to investigate the structural evolution and hydration products of CGSP-containing cement pastes [42]. Figure 19 presents the FTIR spectra of specimens containing different fractions after 3 and 28 d of curing. The absorption band around 3650 cm^−1^ is assigned to the O-H stretching vibration of CH, while the broad band near 3430 cm^−1^ corresponds to the stretching vibration of structural water in C-S-H gel and AFt [42,43]. The band at 1630 cm^−1^ is related to the bending vibration of H-O-H in absorbed water [44]. The peaks at 1420 cm^−1^ and 870 cm^−1^ are attributed to the stretching and bending vibrations of CO_3_^2−^ groups, respectively, indicating the presence of carbonate phases [59,60]. The band around 970 cm^−1^ is associated with Si-O stretching vibrations in C-S-H gel [42], reflecting the formation of calcium silicate hydrate. The absorption near 455 cm^−1^ corresponds to the bending vibration of Si-O-Si bonds from quartz and aluminosilicate structures [61,62,63].

**Figure 20:**
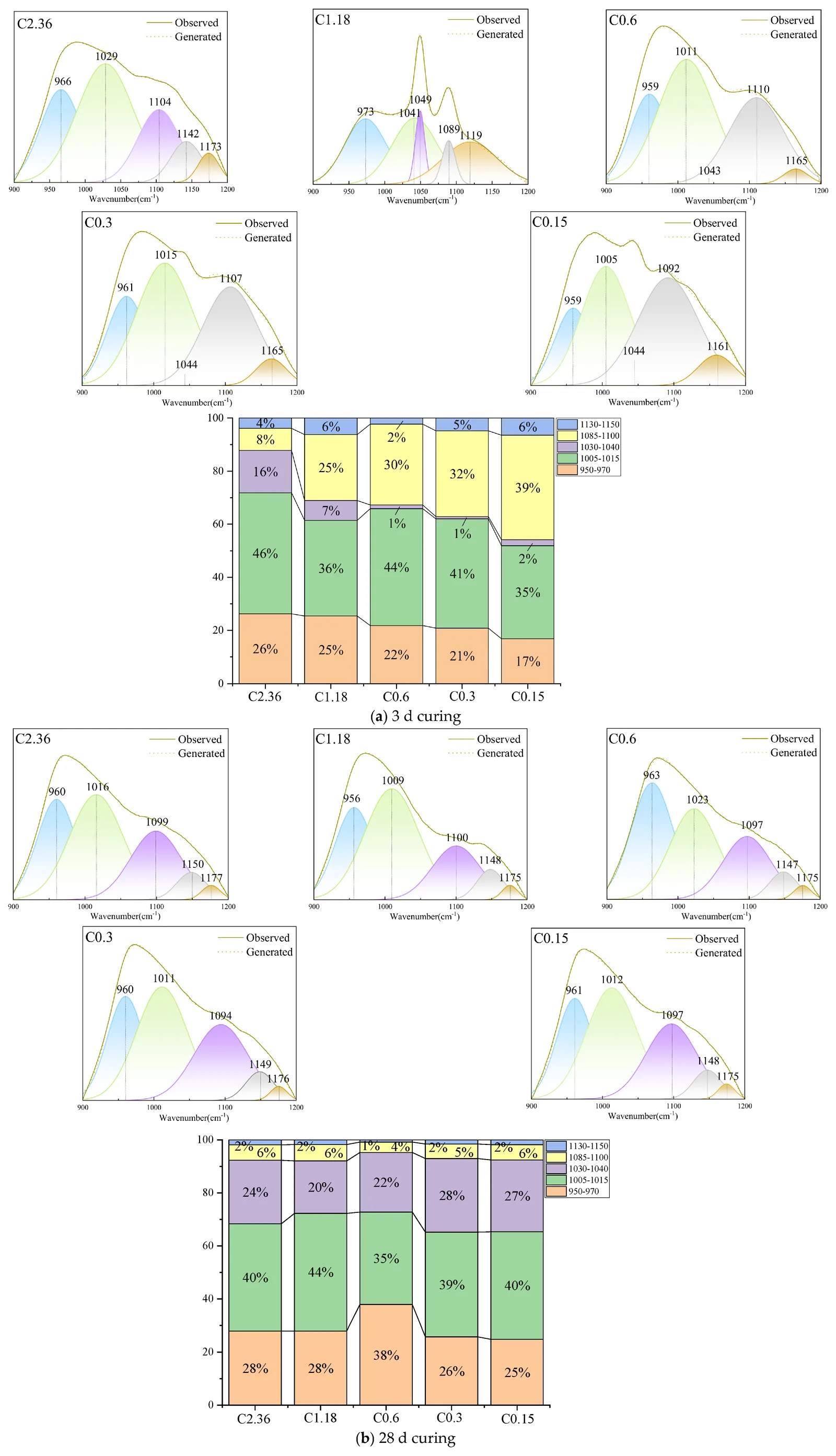
Relative area of deconvoluted peaks in the 900–1200 cm^−1^ region.

To further investigate the polymerization degree of the aluminosilicate framework, deconvolution fitting was performed in the 900–1200 cm^−1^ region (Figure 20). The band at 950–970 cm^−1^ is assigned to the Si-O stretching vibration of Q^1^/Q^2^ silicate units in C-S-H gel, representing the formation of low-polymerized silicate chains [64,65]. The band at 1005–1015 cm^−1^ is mainly associated with the Si-O stretching vibration of C-(A)-S-H gel and partially overlaps with the shoulder vibration of residual C_3_S [66]. The absorption band at 1030–1040 cm^−1^ corresponds to highly polymerized Q^3^ silicate units derived from residual aluminosilicate glass phases [67,68]. The bands located at 1085–1100 cm^−1^ are likely attributed to Q^3^/Q^4^ structures associated with crystalline quartz and unreacted glassy phases, indicating highly polymerized silicate networks. The weak band at 1130–1150 cm^−1^ is related to highly polymerized silicate structures and residual crystalline phases.

From the 3d FTIR spectra (Figure 19a), the C2.36 mixture exhibited the strongest band at approximately 970 cm^−1^. The deconvolution results (Figure 20a) showed a higher contribution of Q^1^/Q^2^ silicate structures associated with C-S-H gel and C-(A)-S-H components, indicating accelerated formation of low-polymerized hydration gels [53]. This may suggest that the reactive glass phase in C2.36 was effectively activated at an early age, contributing to its highest 3d compressive strength.

The C0.6 mixture showed a slightly weaker 970 cm^−1^ band but a stronger broad band near 3430 cm^−1^, which may indicate the formation of more hydrated phases. Combined with the presence of Q^1^/Q^2^ structures, this may suggest that its reactive glass phase had started to participate in hydration, explaining its relatively high early strength [69]. In contrast, the weak 970 cm^−1^ band and enhanced Si-O vibration near 455 cm^−1^ in C0.3 and C0.15 may indicate insufficient formation of C-S-H gel and the presence of more unreacted silicate structures [70].

At 28 d (Figure 19b), the C0.6 mixture exhibited the lowest intensity of the CH-related band at 3650 cm^−1^, which may suggest enhanced pozzolanic reaction and greater CH consumption. Meanwhile, the increased intensity of the 970 cm^−1^ band and higher contribution of C-(A)-S-H components confirmed the formation of abundant hydration gels, which was consistent with its highest 28 d compressive strength [71,72]. Compared with C0.6, the C2.36 mixture showed weaker C-S-H-related bands at later ages, possibly because the rapid early reaction consumed part of the available reactive phases and reduced the contribution of subsequent hydration. The limited changes in characteristic bands of C0.3 and C0.15 further demonstrate their low reactivity and insufficient hydration product formation.

(3)  TG-DTG

**Figure 21:**
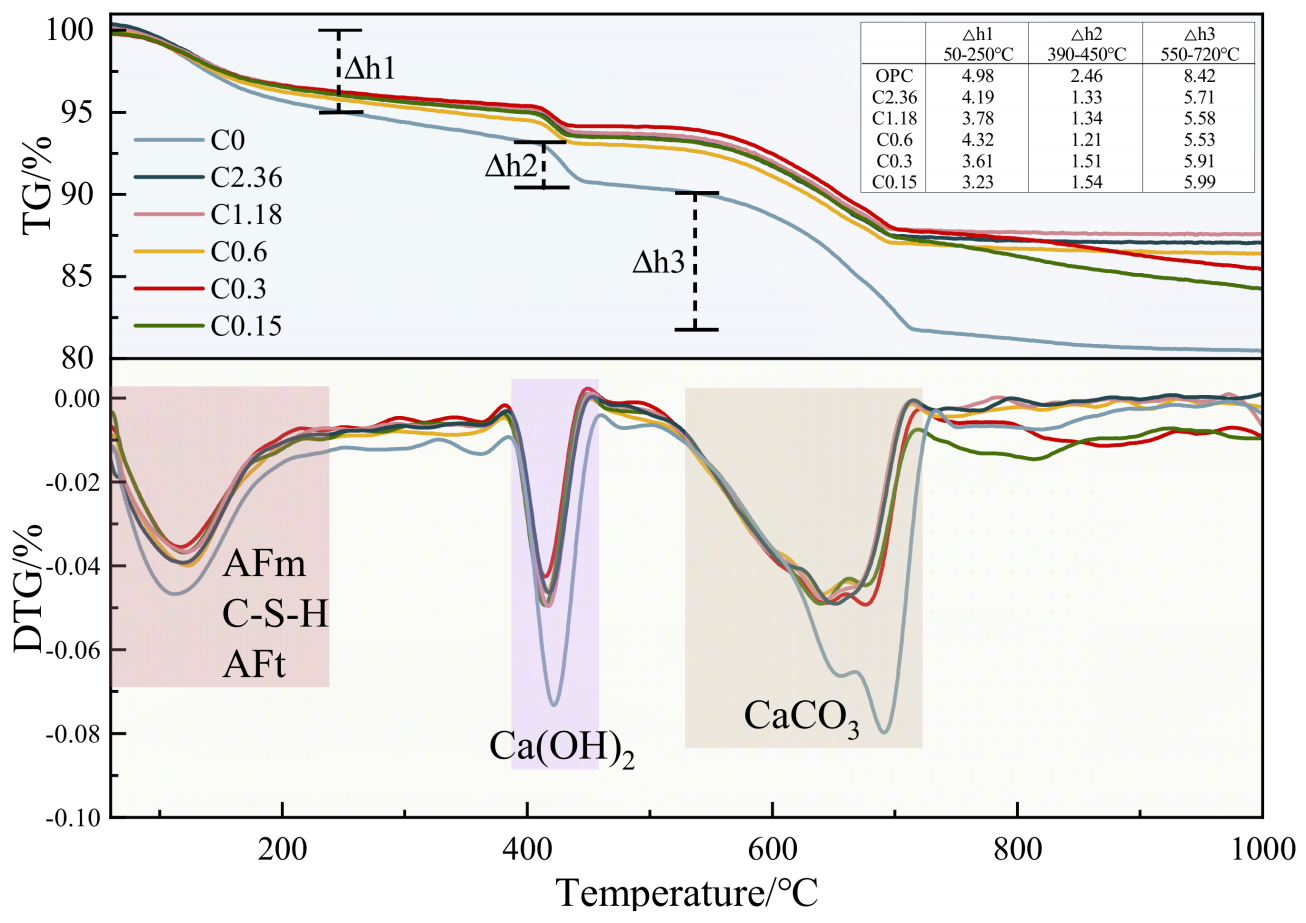
TG-DTG curves of specimens at 28 d of curing.

TG-DTG was performed to evaluate the hydration products of CGSP-containing pastes after 28 d of curing. As shown in Figure 21, all samples exhibited three main mass-loss stages. The first stage (50–250 °C) corresponds to the dehydration of C-S-H gel, AFt, and AFm phases, reflecting the amount of hydrated gel products [73,74,75]. The second stage (390–450 °C) is attributed to the decomposition of CH, while the third stage (550–720 °C) corresponds to the decomposition of CaCO_3_ [76]. Therefore, the mass loss in the first two stages can be used to evaluate the degree of hydration and pozzolanic reaction [76].

Among all mixtures, the C0.6 exhibited the highest mass loss in the first region and the lowest CH decomposition loss, which may suggest the formation of abundant hydration products and extensive CH consumption through pozzolanic reactions. This result confirms that the silica-rich glass phase in C0.6 maintained high reactivity at later ages, promoting continuous C-(A)-S-H gel formation and contributing to its highest 28 d strength [4]. In contrast, C0.15 showed a weak dehydration peak but a relatively high carbonate decomposition loss, which may suggest that its mass loss mainly originated from inert carbonate phases rather than hydration products [77]. The limited formation of C-(A)-S-H gel, which may be associated with the high residual carbon and inert mineral enrichment, appears to have contributed to the poor strength development [58]. The TG-DTG results are consistent with the XRD and FTIR observations.

(4)  SEM-EDS

**Figure 22:**
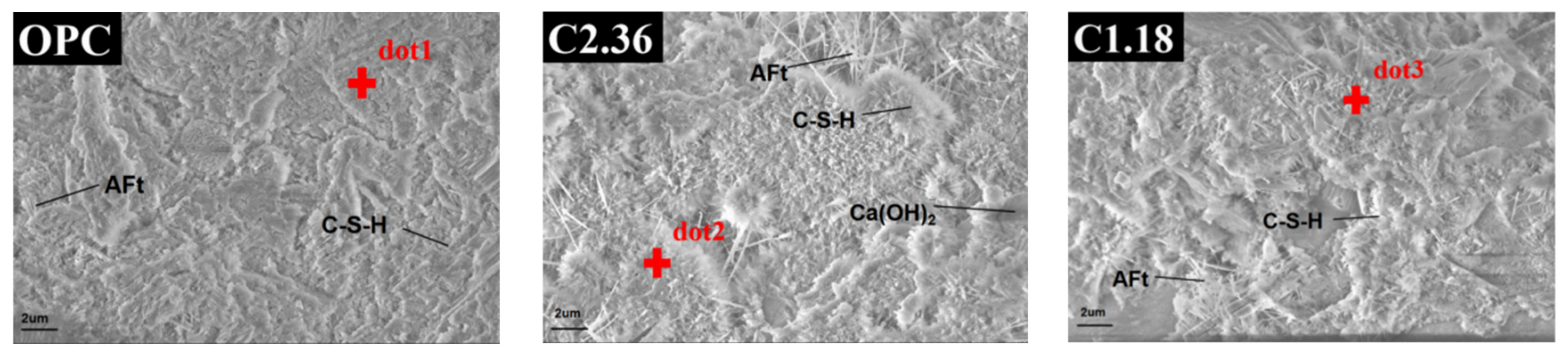

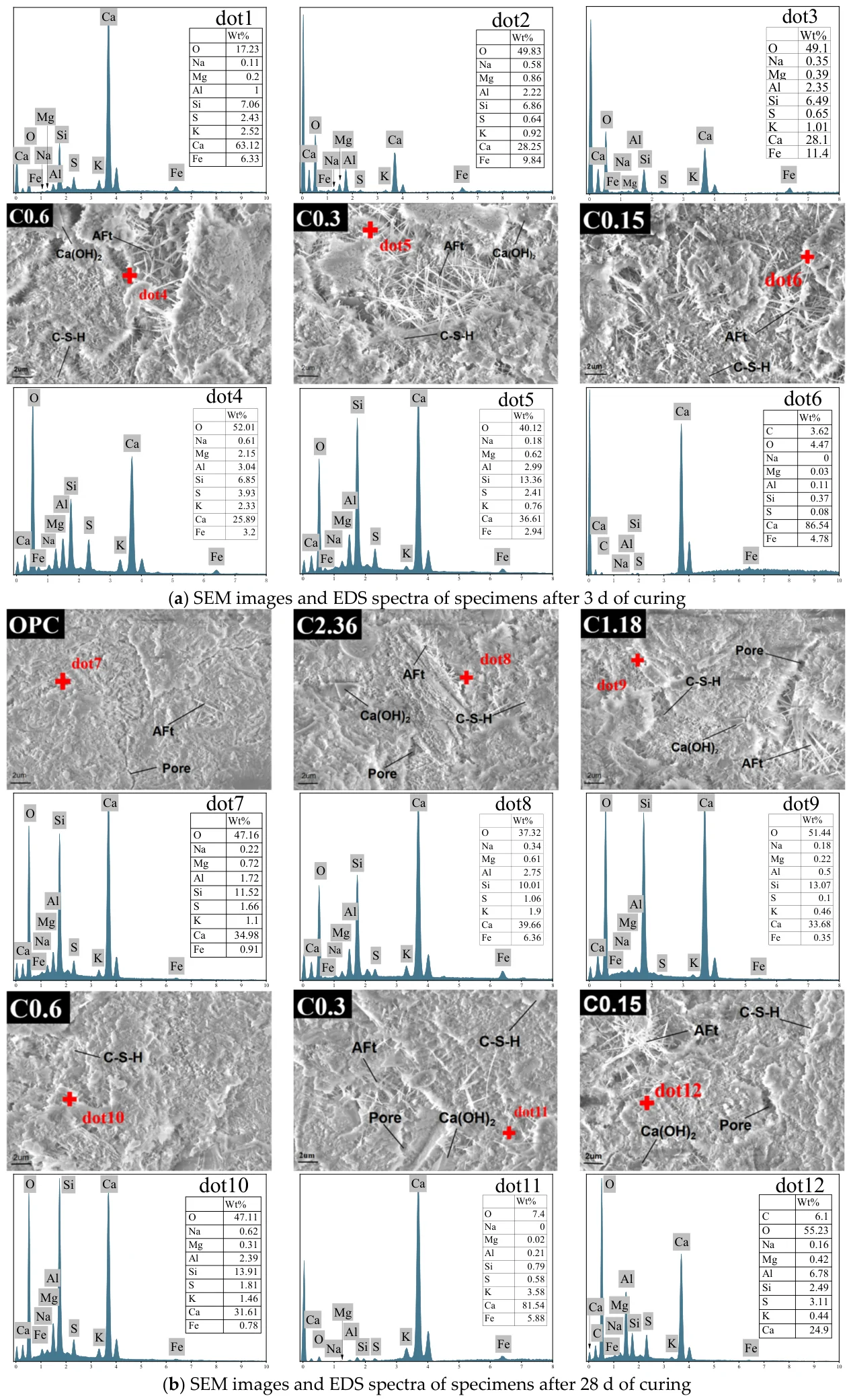
SEM-EDS images of specimens after 3 and 28 d of curing.

Figure 22 presents the SEM images of the OPC paste and CGSP-containing specimens after 3 d and 28 d of curing. The typical hydration products, including needle-like ettringite (AFt), plate-like portlandite (CH), and flocculent C-S-H gel, were observed in all specimens [21]. The increased amount of C-S-H gel in CGSP-containing mixtures indicates the participation of CGSP in secondary hydration reactions, which is consistent with the XRD, FTIR, and TG results.

At 3 d, the C2.36 exhibited the densest microstructure, with abundant C-S-H gel and AFt crystals filling the internal pores. This compact structure may explain its highest early-age strength [24]. The C0.6 showed fewer AFt crystals but visible C-S-H gel formation, suggesting that its reactive glass phase may have begun to dissolve and contribute to hydration, although the reaction rate was relatively slower at the early stage [24].

After 28 d, the C0.6 specimen exhibited the most favorable microstructure among all mixtures. A continuous C-S-H gel network was formed, accompanied by reduced CH crystals and fewer visible pores [3]. The dense hydration products not only refined the capillary pore structure but also improved the interfacial transition zone (ITZ) between CGSP particles and the cement matrix, leading to a stronger and more homogeneous microstructure. This explains its highest 28 d compressive strength. In contrast, the C0.15 specimen contained abundant unreacted particles, residual CH, and larger pores, suggesting insufficient formation of hydration gels. This behavior can be attributed to its high residual carbon content and inert mineral enrichment, which hinder water availability and restrict the growth of hydration products, resulting in poor mechanical performance [58].

## 4. Discussion

**Figure 23:**
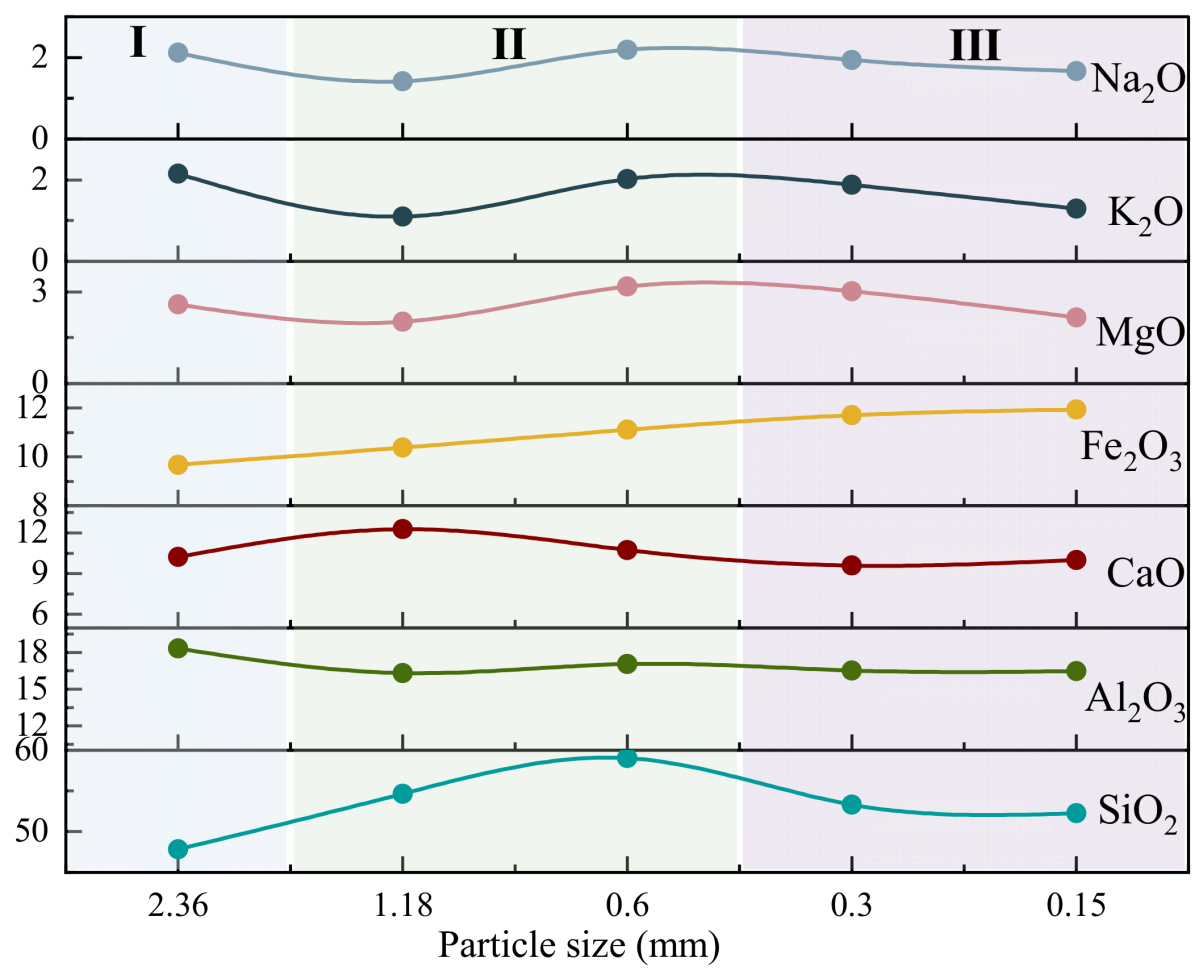
Chemical composition of size-fractionated CGSP.

**Figure 24:**
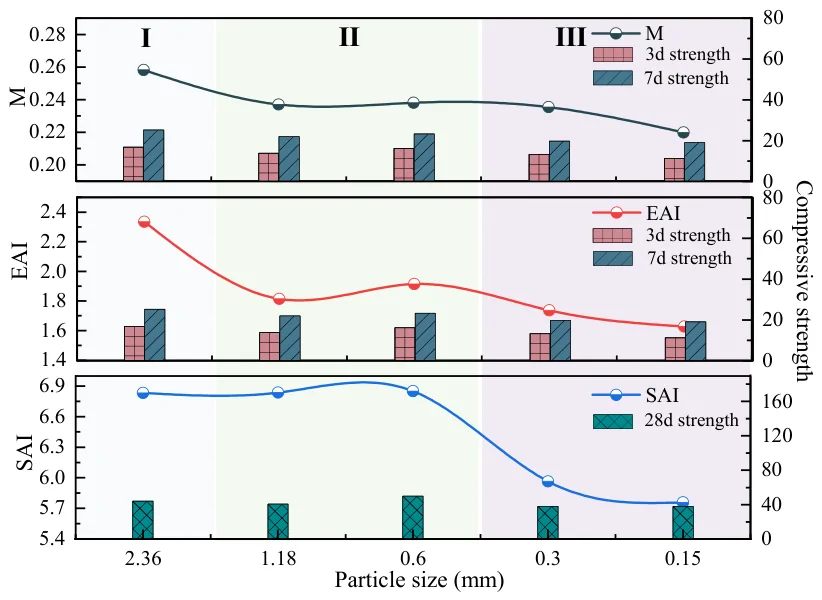
Properties of size-fractionated CGSP.

To further clarify the relationship between the chemical composition of CGSP and strength development, the chemical compositions of different CGSP fractions were compared (Figure 23). Based on previous studies on the chemical evaluation of supplementary cementitious materials, three empirical composition-based parameters were introduced in this study to comparatively assess the potential reactivity of different CGSP fractions, namely the basicity modulus (M), effective activity index (EAI), and silica-alumina activity index (SAI), were introduced, and the corresponding results are presented in Figure 24 [61,78,79,80,81,82]. The M parameter reflects the relative proportion of basic oxides (CaO, MgO, K_2_O, and Na_2_O) to SiO_2_ and Al_2_O_3_, drawing on the concept of slag basicity commonly used in the chemical characterization of supplementary cementitious materials [79]. The EAI and SAI were formulated in this study to compare the relative proportions of Al-containing/alkali components and Si-Al components, respectively, with Fe_2_O_3_. This approach is supported by previous studies showing that SiO_2_, Al_2_O_3_, and Fe_2_O_3_ are important compositional factors related to pozzolanic activity [83]. These parameters are intended as empirical composition-based descriptors rather than standardized measurements of pozzolanic activity or hydration kinetics; therefore, their relationships with compressive strength should be interpreted as comparative correlations within the investigated CGSP fractions.

(1) M = (CaO + MgO + K_2_O + Na_2_O)/(SiO_2_ + Al_2_O_3_)

(2) EAI = (Al_2_O_3_ + K_2_O + Na_2_O)/Fe_2_O_3_

(3) SAI = (SiO_2_ + Al_2_O_3_)/Fe_2_O_3_

According to particle size, the CGSP fractions were divided into three groups: Group I (2.36 mm), Group II (1.18–0.6 mm), and Group III (0.3–0.15 mm). As shown in Figure 23, particle-size separation resulted in significant chemical heterogeneity among different CGSP fractions.

The calculated M and EAI values exhibited a clear relationship with early-age strength development (Figure 24). Group I showed the highest M and EAI values, indicating a stronger alkaline activation capacity and a higher proportion of reactive Al-containing components [82]. These characteristics may promote the depolymerization of the glass network and accelerate the formation of early hydration products [78]. Therefore, Group I exhibited the highest early-age compressive strength, suggesting that M and EAI can effectively reflect the early hydration potential of CGSP. In contrast, the influence of SAI on later-age strength development was more pronounced. As shown in Figure 24, the variation trend of SAI was highly consistent with that of 28 d compressive strength. The higher SAI values of Group II, particularly the 0.6 mm fraction, indicate a higher proportion of reactive Si-Al components and a lower contribution of Fe-containing inert phases. This favorable chemical composition promoted continuous pozzolanic reactions and the formation of C-(A)-S-H gel, resulting in the highest 28 d strength of the 0.6 mm fraction [84]. Group III exhibited lower M, EAI, and SAI values, indicating a deficiency of reactive components and an increased proportion of inert phases. Meanwhile, the enrichment of residual carbon in the finer fractions may have further restricted the dissolution of active glass phases and the growth of hydration products, potentially contributing to limited strength development.

Overall, Figure 23 and Figure 24 demonstrate that the chemical heterogeneity induced by particle-size separation plays an important role in controlling the hydration activity and strength evolution of CGSP. The early-age strength was mainly governed by the initial activation capability represented by M and EAI, whereas the later-age strength was more closely associated with the sustained pozzolanic potential reflected by SAI.

It should be emphasized that M, EAI, and SAI are empirical descriptors proposed for the present study and do not directly quantify the extent or kinetics of hydration reactions. Their relationships with compressive strength should therefore be interpreted as correlations observed within the investigated particle-size fractions, rather than as universal causal relationships.

## 5. Conclusions

This study prepared CGSPs by systematic sieving and grinding of CGS fractions with different particle size ranges. The CGSP was used to replace 40% of Portland cement by mass equivalence. The effects on the workability, mechanical properties, and microstructure of cement paste were systematically investigated. The main conclusions are as follows:

1. Significant particle-size-dependent heterogeneity was observed in the physicochemical characteristics of CGS. With decreasing particle size, the water absorption, residual carbon content, Fe_2_O_3_ content, and density generally increased, whereas the crushing value decreased. Meanwhile, considerable variations in mineral composition, glassy phase content, and reactive component distribution were observed among different fractions, indicating the intrinsic heterogeneity of CGS.
2. The incorporation of CGSP significantly delayed the setting process of cement paste, with the initial and final setting times extended by 26.9–52.6% and 27.0–32.2%, respectively.
3. Except for the C0.15 fraction, which was negatively affected by its high residual carbon content and water demand, the addition of CGSP improved paste fluidity by 12.6–35.2%, mainly due to the micro-filling effect and particle lubrication effect.
4. Different CGSP fractions exhibited distinct effects on strength development. The C2.36 fraction showed the highest early-age strength due to its favorable chemical composition and enhanced early hydration activity, which promoted the rapid formation of hydration products. In contrast, the C0.6 fraction demonstrated superior later-age strength development owing to its higher reactive silica-rich glass phase, which sustained the pozzolanic reaction and promoted additional C-(A)-S-H gel formation. The C0.15 fraction exhibited the poorest mechanical performance at all ages, which may be attributed to its high Fe_2_O_3_ and residual carbon contents and limited reactive phase availability.
5. Microstructural analysis confirms the consistency between hydration degree and strength development. C2.36 exhibited the highest amount of early hydration products (AFt and C-S-H), resulting in a dense structure. C0.6 showed sufficient CH consumption at later ages, with C-S-H gel becoming the dominant hydration product and forming a homogeneous and dense matrix. In contrast, C0.15 contained fewer hydration products and a more porous and loose structure. Overall, the degree of hydration is consistent with the observed strength development trends across all specimens.
6. At the investigated 40 wt.% replacement level, the results demonstrate the potential of a “graded grinding and fraction-specific utilization” strategy. In practical applications, the selection of CGS fractions should be tailored according to engineering requirements for either early-age or long-term strength. The 2.36–4.75 mm fraction is more suitable when early performance is prioritized, whereas the 0.6–1.18 mm fraction shows greater potential for long-term performance.

## Figures and Tables

**Table 1:** Main chemical composition of PO·42.5 and CGS (wt%).

Oxide	SiO_2_	Al_2_O_3_	CaO	Fe_2_O_3_	MgO	K_2_O	Na_2_O
CGS	52.3	16.5	9.9	10.5	2.5	1.7	1.6
PO·42.5	18.8	5.68	63.59	3.38	1.78	1.07	0.211

**Table 2:** Specific surface area of fractionated CGSP (m^2^/g).

Particle Size	2.36 mm (2.36–4.75 mm)	1.18 mm (1.18–2.36 mm)	0.6 mm (0.6–1.18 mm)	0.3 mm (0.3–0.6 mm)	0.15 mm (0.15–0.3 mm)
Specific surface area	0.289	0.281	0.230	0.338	0.255

**Table 3:** Mix proportions.

No.	OPC/g	CGSP/g	Water/g
C0	700	0	252
C2.36	420	280	252
C1.18	420	280	252
C0.6	420	280	252
C0.3	420	280	252
C0.15	420	280	252

**Table 4:** Characteristics of CGS with different particle sizes.

Particle Size	Bulk Density (g/cm^3^)	Apparent Density (g/cm^3^)	Porosity (%)	Water Absorption (%)	LOI (%)
2.36 mm (2.36–4.75 mm)	1.31	2.52	48.02	5.67	0.12
1.18 mm (1.18–2.36 mm)	1.26	2.53	50.20	6.96	0.18
0.6 mm (0.6–1.18 mm)	1.34	2.54	47.24	10.59	0.22
0.3 mm (0.3–0.6 mm)	1.35	2.51	46.22	17.99	0.32
0.15 mm (0.15–0.3 mm)	1.19	2.53	52.96	30.30	1.13

**Table 5:** Chemical composition of fractionated CGSP (wt.%).

	SiO_2_	Al_2_O_3_	CaO	Fe_2_O_3_	MgO	K_2_O	Na_2_O
2.36 mm (2.36–4.75 mm)	47.82	18.33	10.22	9.68	2.59	2.15	2.12
1.18 mm (1.18–2.36 mm)	54.65	16.31	12.28	10.38	2.02	1.10	1.42
0.6 mm (0.6–1.18 mm)	59.04	17.06	10.74	11.11	3.17	2.02	2.19
0.3 mm (0.3–0.6 mm)	53.32	16.52	9.60	11.71	3.02	1.88	1.94
0.15 mm (0.15–0.3 mm)	52.27	16.48	10.00	11.94	2.16	1.29	1.67

## Data Availability

The original contributions presented in this study are included in the article. Further inquiries can be directed to the corresponding author.
